# A novel nomogram for predicting osteoporosis with low back pain among the patients in Wenshan Zhuang and Miao Autonomous Prefecture of China

**DOI:** 10.3389/fendo.2025.1535163

**Published:** 2025-06-05

**Authors:** Man Jia, Qinzuo Dong, Zeqin Ren, Liwei Xing, Jinjie Li, Xiaomei Wang, Shun Yu, Xiaoyan Wang, Rong Zhao

**Affiliations:** ^1^ The First Clinical Medical College, Nanjing University of Chinese Medicine, Nanjing, Jiangsu, China; ^2^ Acupuncture Department and Department of Information Technology, People's Hospital of Wenshan Zhuang and Miao Autonomous Prefecture, Wenshan, Yunnan, China; ^3^ First Clinical Medical College, Yunnan University of Chinese Medicine, Kunming, Yunnan, China; ^4^ Department of Rehabilitation, The First Affiliated Hospital of Dali University, Dali, Yunnan, China; ^5^ Acupuncture and Moxibustion Department, Yuxi Municipal Hospital of Traditional Chinese Medicine, Yuxi, Yunnan, China

**Keywords:** low back pain, osteoporosis, nomogram, predicting, Autonomous Prefecture

## Abstract

**Background:**

Low back pain (LBP) is one of the most common symptoms of osteoporosis (OP), but LBP caused by osteoporosis can easily be masked by other causes, leading to misdiagnosis. However, there are currently no convenient tools available to identify patients with low back pain caused by osteoporosis.

**Methods:**

We consecutively enrolled 769 patients diagnosed with low back pain in our hospital from January 2019 to March 2024. A total of 355 cases were excluded due to relevant missing data, leaving a final analysis cohort of 414 cases. The dataset was randomly divided into a training group and a validation group at a ratio of 7:3 for further analysis. in this preliminary analysis were selected for subsequent multivariate analysis. Least absolute shrinkage and selection operator(LASSO) was employed to identify the associated risk factors for osteoporosis. Independent variables with P<0.05 in univariate analysis were included in the multivariate analysis to construct the prediction model. Once the regression equation was established, a nomogram was utilized to visualize the prediction model, while receiver operating characteristic (ROC) curve was plotted to evaluate its performance, specifically by calculating the area under the curve (AUC) which represents discrimination ability of the model. To assess goodness-of-fit, calibration curve was generated for evaluating calibration accuracy. Furthermore, decision curve analysis (DCA) served to determine clinical application value of this predictive model. Statistical significance level was set at P < 0.05.

**Results:**

Building upon the LASSO and multivariate Cox regression, eleven variables were significantly associated with OP (i.e., gender, age, history of fracture, history of alcohol consumption, history of rheumatoid arthritis, hematocrit, red blood cell volume distribution width, lymphocyte percentage, triglyceride, potassium ion, and alanine aminotransferase). In training and validation sets, AUCs and C-indexes of the OP prediction models were all greater than 0.8(AUC: 0.914 for training; 0.833 for validation), which indicated excellent predictability of models. On the whole, the calibration curves coincided with the diagonal in two models. DCA indicated that the models had higher clinical benefit than other risk factors. While confirmed the clinical utility of the model, as it outperformed both the ‘treat-all’ and ‘treat-none’ strategies.

**Conclusion:**

After verification, our prediction models of OP are reliable and can predict the incidence of osteoporosis, providing valuable guidance for clinical prognosis estimation and individualized administration of patients with LBP(a new way for early identification and intervention of patients with osteoporosis).

## Introduction

Low back pain (LBP) is a major global health issue with significant epidemiological and economic implications. Its prevalence is 7.3%, affecting approximately 540 million people daily (1). Furthermore, LBP is a leading cause of disability worldwide (2), and recent evidence suggests that up to 32% of patients with acute back pain progress to chronic low back pain (CLBP). Chronic LBP (CLBP) can lead to long-term disability and decreased quality of life (3). Osteoporosis (OP), which manifests as acute or chronic persistent low back pain, is closely associated with CLBP, especially in postmenopausal women (4). Osteoporosis progresses slowly, often going unnoticed until a fracture occurs. Low back pain, however, is frequently the first clinical symptom of osteoporosis (5). Therefore, early prediction of osteoporosis in patients with low back pain is essential. Effective risk stratification and management strategies for chronic LBP could prevent osteoporosis-related events, improving patient outcomes.

Osteoporosis (OP) is characterized by reduced bone mineral density (BMD) and an increased risk of fractures (6, 7). Its prevalence ranks just behind cardiovascular diseases and diabetes (8–10), making it the third most common endocrine and metabolic disease after diabetes and thyroid disorders (11–13). By 2050, it is estimated that 212 million people worldwide will be diagnosed with osteoporosis (14–16). Osteoporotic fractures significantly impact the lives of affected individuals, decreasing both personal and family quality of life (17). Vertebral and hip fractures, in particular, are associated with increased mortality risk (18). Osteoporosis has an insidious onset, often presenting with leg cramps and pain, with typical symptoms including back and hip pain (19). It is frequently misdiagnosed, leading to delays in diagnosis and treatment. Early detection, coupled with timely interventions and effective strategies for prevention, treatment, and management, can significantly reduce osteoporosis-related adverse events (20). Dual-energy X-ray absorptiometry (DXA) remains the gold standard for osteoporosis diagnosis (21), but its high cost and radiation exposure limit its use for routine or repeated assessments (22).

Wenshan Zhuang and Miao Autonomous Prefecture, located in the southeastern part of Yunnan Province, China, shares a border with Vietnam. The mountainous and semi-mountainous regions cover 97% of its total area, and the border stretches over 438 kilometers. The region is home to numerous ethnic minorities, including Zhuang, Miao, Yi, Yao, and Hui, who make up more than half of the total population. The area is characterized by remote geographical conditions, widespread poverty, and limited access to healthcare, making it a minority poverty-stricken area (Information sourced from the official website of Wenshan Zhuang and Miao Autonomous Prefecture People’s Government, accessed on November 17, 2024, at 14:38). Due to geographical isolation, altitude, and lifestyle factors, the minority populations in this region are more likely to overlook the harmful effects of chronic pain, and limited healthcare resources exacerbate this issue. Osteoporosis-related fractures due to low back pain are often neglected in this region (23). Consequently, there is a pressing need for a safe, simple, and practical screening method or predictive model to replace DXA for bone density testing in these populations.

Several studies have shown that low hemoglobin levels may increase the risk of osteoporotic fractures in elderly men (24). A study investigating the relationship between the monocytic-to-lymphocytic ratio (MLR) and osteoporosis in postmenopausal women with type 2 diabetes in China revealed that MLR has high diagnostic efficacy for osteoporosis and may serve as a biomarker for diagnosing osteoporosis in this population (25). Research from Guangzhou University of Chinese Medicine indicated that low hemoglobin levels are associated with a higher risk of osteoporotic fractures in elderly men (26). A study conducted in Jiangsu Province found that the uric acid/high-density lipoprotein cholesterol ratio (UHR) is an independent risk factor for abnormal bone mineral density in elderly diabetic patients (27). A cross-sectional study from Sun Yat-sen University, Guangzhou, China, showed that plasma fibrinogen levels are negatively correlated with BMD in hypertensive patients, suggesting that plasma fibrinogen could serve as a potential screening marker for osteoporosis, aiding early diagnosis and monitoring treatment (28). Erythrocyte distribution width (RDW) may indirectly affect bone metabolism and contribute to the development of osteoporosis through its association with factors such as inflammatory response, anemia, oxidative stress, and microcirculatory function, Platelets contribute to bone metabolism through multiple mechanisms, including the secretion of cytokines, modulation of osteoclast activity, and involvement in immune regulation (29).

The studies referenced indicate a correlation between osteoporosis and various hematological indicators, including standard blood parameters such as the MLR, hemoglobin levels, and fibrinogen, as well as biochemical parameters like the UHR. In the Wenshan region, these associations are particularly pronounced due to its distinct epidemiological context, characterized by a higher prevalence of thalassemia compared to non-minority regions and limited access to BMD testing equipment. This combination of genetic predisposition, such as thalassemia-related anemia, and inadequate diagnostic infrastructure presents significant challenges in addressing osteoporosis-related health disparities within this underserved population. This study seeks to create a practical model to predict osteoporosis in low back pain patients by combining the monocyte-to-lymphocyte ratio (MLR) with standard clinical and lab parameters, tailored for resource-limited areas like the Wenshan Zhuang and Miao Autonomous Prefecture. The proposed study aims to investigate the association between various blood markers—specifically hemoglobin levels, the lymphocyte-to-monocyte ratio, uric acid, high-density lipoprotein cholesterol, low-density lipoprotein cholesterol, and plasma fibrinogen—and the incidence of osteoporosis within this region. Data will be collected and analyzed from a cohort of 769 patients experiencing low back pain to identify factors that contribute to the onset of osteoporosis. A predictive model will be constructed to examine the correlation between routine blood test results and the prevalence of osteoporosis. The findings of this research are expected to facilitate the early detection of osteoporosis and inform the development of effective management and treatment strategies. Ultimately, the study aims to enhance the quality of life for the elderly population in the region and to mitigate the risk of bone-related injuries.

## Materials and methods

Participant Population: The clinical data of patients with LBP (Identification of LBP in the Hospital Information System.), includes op patients(The diagnosis is confirmed by bone mineral density measured using Dual-energy X-ray Absorptiometry with T-scores≤-2.5 SD at either the lumbar spine (L1-L4) or femoral neck, following WHO (1994) criteria.”.) admitted to the People’s Hospital of Wenshan Zhuang and Miao Autonomous Prefecture between January 2019 and March 2024 were retrospectively reviewed. The research protocol was approved by the hospital’s(The People’s Hospital of Wenshan Zhuang and Miao Autonomous Prefecture in Yunnan province.) ethics committee (project number: WYLS 2024005, ethical review number: WYLS2024‐005). Informed consent was waived due to the retrospective use of anonymized data.

### Data collection

Clinical data were extracted from electronic medical records using a standardized form. Two independent researchers entered the data, resolving any discrepancies by consensus.

The inclusion criteria were as follows:

1. Patients with evident low back pain and restricted physical activity, particularly in cases involving turning over or getting up.
2. Patients with chronic low back pain admitted to the People’s Hospital of Wenshan Zhuang and Miao Autonomous Prefecture during the study period.
3. Patients who had resided in their current location for more than five years.
4. omplete clinical data were available.
5. Patients demonstrated good compliance with the study protocol.

The exclusion criteria were as follows:

1. Patients who did not present with low back pain.
2. Patients not originating from the Wenshan region.
3. Patients with congenital bone anomalies.
4. Patients with anatomical variations, bone tumors, or osteonecrosis.
5. Incomplete follow-up data and poor patient compliance were also exclusion factors.

### Variables

The study retrospectively analyzed the clinical data and biological indicators of enrolled patients, focusing on demographic characteristics, disease history, and inflammatory and metabolic indicators, included 47 variables.

### Statistical analysis

Statistical analyses were conducted using SPSS version 26.0 and R language version 4.4.1. Quantitative data following a normal distribution were expressed as means ± standard deviations, while non-normally distributed data were reported as medians with interquartile ranges. Categorical data were presented as frequencies or percentages. Continuous variables were analyzed using t-tests or U-tests, while categorical variables were assessed using chi-square tests or Fisher’s exact tests. The dataset was randomly split into training and validation groups in a 7:3 ratio. The significance of each variable in the training cohort was assessed by univariate logistic regression analysis for investigating the independent risk factors of presence of OP. We first conducted univariate logistic regression to examine the individual association between each potential predictor and the risk of osteoporosis. Variables that demonstrated statistical significance (*p* < 0.05) in this preliminary analysis were selected for subsequent multivariate analysis. Significant variables identified in the univariate analysis were then included in the multivariate logistic model using both stepwise forward and backward selection methods. Variables with *p* < 0.05 were retained in the final model, while non-significant predictors were sequentially excluded. The model’s performance was assessed using the AUC. Given that the forward selection method resulted in a more favorable AIC and included fewer independent variables compared to the backward elimination method (Full candidate variables are listed in **Supplementary Table S3**), the final model was constructed using the forward selection approach. LASSO was applied to evaluate risk factors associated with osteoporosis, with odds ratios (OR) and 95% confidence intervals (CI) calculated. Independent variables with *p* < 0.05 were included as predictors in the multivariate analysis to construct the predictive model. Once the regression equation was established, the predictive model was visualized using nomograms, and ROC curves were generated to assess the model’s performance. The AUC was calculated to evaluate the model’s discriminatory power. The model’s goodness-of-fit was tested using the Hosmer-Lemeshow test, and calibration curves were plotted to assess the model’s accuracy. Furthermore, DCA was performed to evaluate the clinical applicability of the model. A significance level of *p* < 0.05 was considered statistically significant.

## Results

### Baseline characteristics

**Figure 1:**
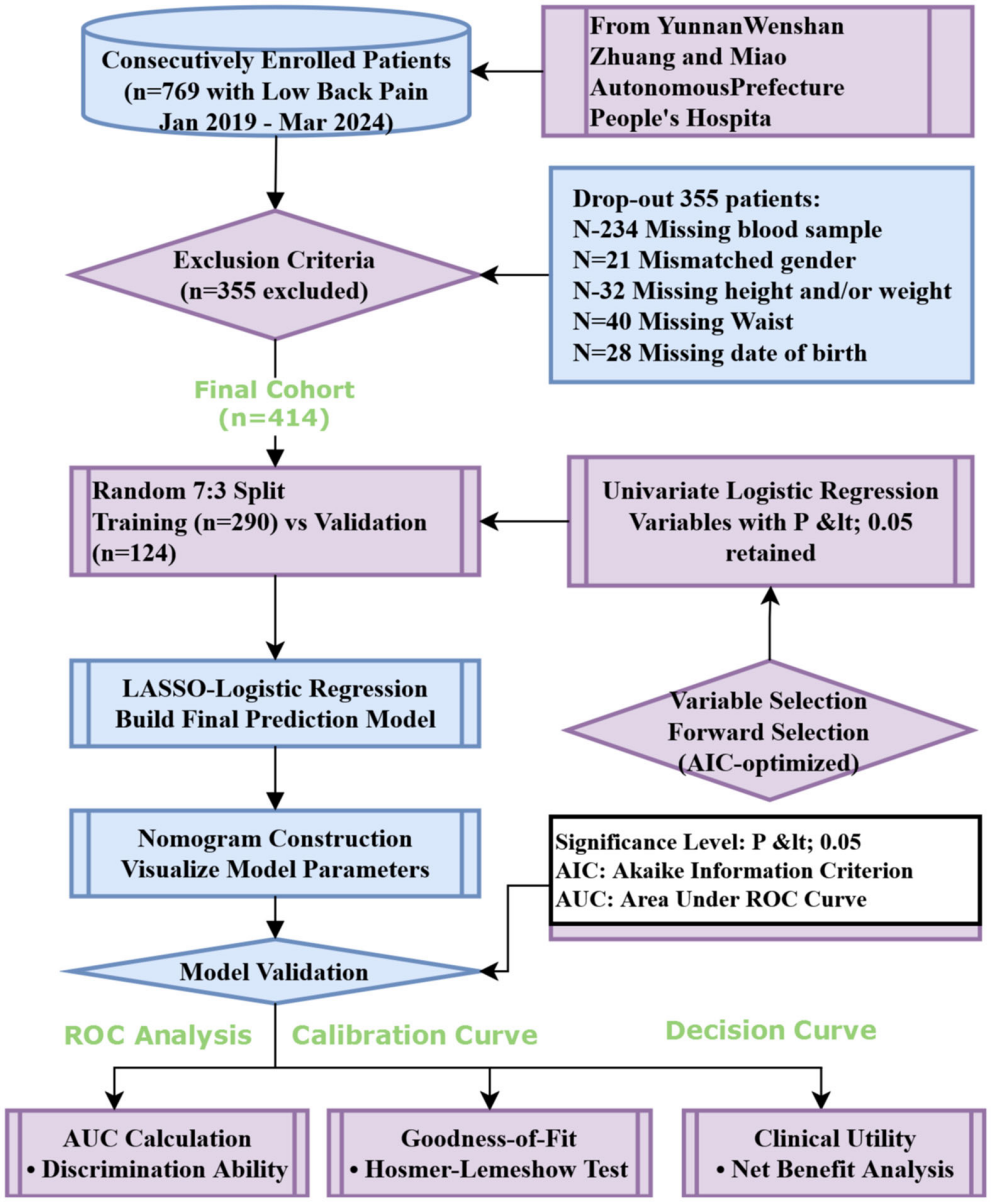
Flow chart.

**Figure 2:**
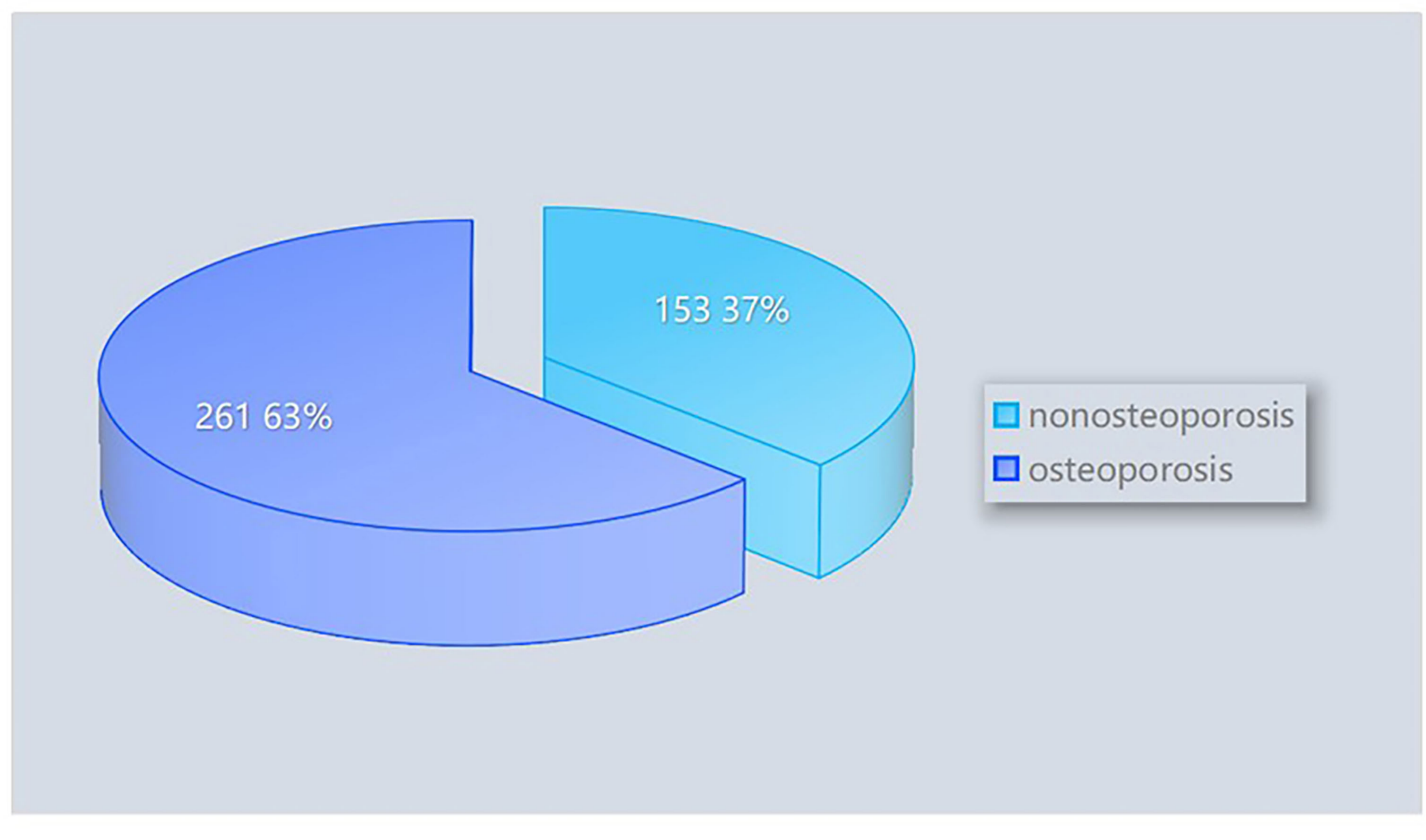
Composition of osteoporosis and non-osteoporosis patients within the total dataset of low back pain patients.

From January 2019 to March 2024, patients diagnosed with LBP in the People’s Hospital of Wenshan Zhuang and Miao Autonomous Prefecture, Yunnan Province, were screened for inclusion. After the screening process, 769 patients with relatively complete clinical data were included in the study. Based on a review of the literature and consideration of local dietary and lifestyle habits, 76 variables were identified for evaluation. Relevant patient information was retrieved and extracted using the Hospital Information System (HIS) based on these variables. A total of 355 cases with incomplete observational variables were excluded, leaving 414 patients with low back pain as the final study cohort. Among these, 261 patients were diagnosed with osteoporosis, while 153 patients were not (**Figure 1**). The distribution of patients with and without osteoporosis within the total dataset of low back pain patients is depicted in **Figure 2**.

Statistical analysis revealed that the mean age of patients in the osteoporosis group was significantly higher than in the non-osteoporosis group (75.00 vs. 62.00 years, *p* < 0.001). Additionally, significant differences were found in several biochemical markers: total cholesterol (4.43 vs. 5.10 mmol/L, *p* < 0.001), low-density lipoprotein (LDL) (2.52 vs. 2.96 mmol/L, *p* < 0.001), creatine kinase isoenzyme (16.00 vs. 19.00 U/L, *p* = 0.001), C-reactive protein (8.60 vs. 3.80 mg/L, *p* < 0.001), hemoglobin (123.00 vs. 139.00 g/L, *p* < 0.001), and glycated hemoglobin (5.49 vs. 5.14 mmol/L, *p* = 0.006). Furthermore, D-dimer levels (1.11 vs. 0.59 mg/L, *p* < 0.001) and neutrophil percentage (72.80% vs. 70.10%, *p* = 0.057) also showed differences.

Regarding gender distribution, 69 males (28.74%) and 75 females (45.10%) were in the osteoporosis group, compared to 84 males (54.90%) and 186 females (71.26%) in the non-osteoporosis group, with a significant difference observed (*p* < 0.001). Other parameters, including systolic and diastolic blood pressure, triglycerides, and serum magnesium, showed no significant differences (*p* > 0.05). In summary, patients with osteoporosis exhibited significant variations in multiple physiological and biochemical parameters compared to those without osteoporosis, suggesting a potential relationship between osteoporosis and changes in these indicators. Detailed data can be found in **Table 1**. (Full candidate variables are listed in **Supplementary Table S1**)

**Table 1:** Baseline characteristics of osteoporosis and non-osteoporosis groups in low back pain patients (N=414).

Category	Variable	Non-Osteoporosis (n=153)	Osteoporosis (n=261)	Statistical Value	P Value
Demographics	Age (years)	62.00 (53.00–73.00)	75.00 (69.00–82.00)	82.601	<0.001
	Sex (Male), n (%)	69 (45.10%)	75 (28.74%)	11.384	<0.001
	Ethnicity (Han), n (%)	114 (74.51%)	195 (74.71%)	30.721	<0.001
Clinical History	Smoking (Yes), n (%)	25 (16.34%)	51 (19.54%)	0.659	0.417
	Drinking (Yes), n (%)	16 (10.46%)	44 (16.86%)	3.189	0.074
	Hypertension (Yes), n (%)	58 (37.91%)	117 (44.83%)	1.892	0.169
	Cerebral Infarction (Yes), n (%)	2 (1.31%)	14 (5.36%)	4.273	0.039
	Encephalatrophy (Yes), n (%)	6 (3.92%)	22 (8.43%)	3.108	0.078
	Fracture (Yes), n (%)	2 (1.31%)	76 (29.12%)	48.792	<0.001
Laboratory	Systolic BP (mmHg)	128.00 (118.00–143.00)	130.00 (118.00–146.00)	1.285	0.257
	Diastolic BP (mmHg)	80.00 (71.00–88.00)	78.00 (70.00–88.00)	0.511	0.475
	Total Cholesterol (mmol/L)	5.10 (4.21–6.03)	4.43 (3.75–5.25)	21.386	<0.001
	LDL (mmol/L)	2.96 (2.25–3.48)	2.52 (1.94–3.11)	18.376	<0.001
	HDL (mmol/L)	1.24 (1.06–1.45)	1.22 (1.03–1.45)	0.514	0.473
	Triglyceride (mmol/L)	1.44 (1.06–2.44)	1.37 (1.05–1.87)	3.061	0.080
	Glucose (mmol/L)	5.14 (4.79–6.03)	5.49 (4.89–6.53)	7.429	0.006
	C-reactive Protein (mg/L)	3.80 (1.40–10.60)	8.60 (3.80–51.60)	28.424	<0.001
	D-dimer (mg/L)	0.59 (0.29–1.94)	1.11 (0.44–2.91)	15.287	<0.001
Hepatic/Renal	Alanine Aminotransferase (U/L)	20.00 (14.00–35.00)	16.00 (9.00–26.00)	15.675	<0.001
	Aspartate Aminotransferase (U/L)	21.00 (18.00–29.00)	23.00 (18.00–32.00)	1.310	0.252
	Alkaline Phosphatase (U/L)	84.00 (71.00–97.00)	87.00 (73.00–102.00)	1.820	0.177
	Albumin (g/L)	39.90 (37.20–42.10)	37.80 (35.10–40.60)	19.133	<0.001
	Creatinine (μmol/L)	67.00 (56.00–82.00)	71.00 (58.00–92.00)	3.665	0.056
Hematology	Hemoglobin (g/L)	139.00 (127.00–149.00)	123.00 (99.00–137.00)	49.906	<0.001
	Red Blood Cell (g/L)	4.62 ± 0.59	4.16 ± 0.66	50.143	<0.001
	Platelet Count (10^9^/L)	248.00 (207.00–301.00)	240.00 (191.00–304.00)	0.332	0.564
	Lymphocyte Count (10^9^/L)	1.93 (1.55–2.21)	1.59 (1.17–2.01)	22.571	<0.001
	Neutrophil Count (10^9^/L)	5.05 (3.63–7.48)	5.25 (3.65–7.40)	0.085	0.771
	White Blood Cell (10^9^/L)	7.31 (5.71–8.84)	7.37 (5.71–8.88)	0.237	0.626
Other Biomarkers	Uric Acid (μmol/L)	329.00 (274.00–400.00)	308.00 (247.00–410.00)	2.170	0.141
	Procalcitonin (ng/ml)	0.04 (0.03–0.10)	0.07 (0.04–0.23)	15.923	<0.001
	Calcium (mmol/L)	2.25 (2.18–2.32)	2.23 (2.15–2.31)	3.213	0.073

Data presented as median (IQR), mean ± SD, or n (%).

Statistical tests: Mann-Whitney U/t-test for continuous variables; Chi-square/Fisher’s exact test for categorical variables.

Significance: P < 0.01.

BP, blood pressure; LDL, low-density lipoprotein; HDL, high-density lipoprotein.

### Data segmentation

In this study, a total of 414 patients were included, with 124 patients assigned to the test cohort and 290 patients to the training cohort. Some patients were excluded due to incomplete or insufficient data. In the test cohort, 77.17% of the patients were male, compared to 71.01% in the training cohort, with a median age of 73 years (IQR 63.75-80.25 years). Comparative analysis of baseline characteristics revealed no significant differences in systolic blood pressure (SBP) or age (p > 0.05). However, there were significant differences in diastolic blood pressure (DBP), triglycerides (TG), magnesium (Mg), and mean platelet volume (MPV) (p < 0.05). Further details are provided in **Table 2**. (Full candidate variables are listed in **Supplementary Table S2**)

**Table 2:** Baseline characteristics of confirmed and unconfirmed osteoporosis patients in training and validation cohorts (N=414).

Category	Variable	Validation (n=124)	Training (n=290)	Statistical value	P value
Demographics	Age (years)	73.00 (63.75–80.25)	72.00 (60.00–79.00)	0.271	0.603
	Sex (Male), n (%)	40 (32.26%)	104 (35.86%)	0.497	0.481
	Ethnicity (Han), n (%)	89 (71.77%)	220 (75.86%)	8.459	0.489
Clinical History	Smoking (Yes), n (%)	17 (13.71%)	59 (20.34%)	2.551	0.110
	Drinking (Yes), n (%)	12 (9.68%)	48 (16.55%)	3.312	0.069
	Hypertension (Yes), n (%)	53(42.74%)	122 (42.07%)	0.016	0.899
	Cerebral Infarction (Yes), n (%)	4 (3.23%)	12 (4.14%)	0.195	0.659
	Encephalatrophy (Yes), n (%)	8 (6.45%)	20 (6.90%)	0.027	0.869
	Fracture (Yes), n (%)	29 (23.39%)	49 (16.90%)	2.393	0.122
Laboratory	Systolic BP (mmHg)	129.00 (119.00-144.50)	130.00 (117.00-146.00)	0.01	0.92
	Diastolic BP (mmHg)	76.50 (67.75-86.00)	80.00 (70.00-89.00)	4.803	0.028
	Total Cholesterol (mmol/L)	4.77 (3.92-5.66)	4.62 (3.91-5.51)	0.706	0.401
	LDL (mmol/L)	2.73 (2.08-3.26)	2.68 (2.07-3.32)	<0.001	0.975
	HDL (mmol/L)	1.22 (1.04-1.41)	1.24 (1.03-1.47)	0.205	0.651
	Triglyceride (mmol/L)	1.52 (1.10-2.27)	1.35 (1.03-2.06)	3.774	0.052
	Glucose (mmol/L)	5.34 (4.86-6.39)	5.36 (4.81-6.33)	0.338	0.561
	C-reactive Protein (mg/L)	7.00 (2.58-34.20)	6.20 (2.24-32.52)	0.586	0.444
	D-dimer (mg/L)	1.00 (0.44-2.84)	0.84 (0.35-2.37)	1.646	0.200
Hepatic/Renal	Alanine Aminotransferase (U/L)	18.00 (11.00-29.25)	17.00 (11.00-28.75)	0.159	0.69
	Aspartate Aminotransferase (U/L)	23.00 (18.00–31.00)	22.00 (18.00–30.00)	0.046	0.83
	Alkaline Phosphatase (U/L)	86.00 (73.75-98.00)	85.50 (71.00-99.75)	0.039	0.843
	Albumin (g/L)	39.15 (36.10-41.20)	38.80 (35.73-41.35)	0.519	0.471
	Creatinine (μmol/L)	68.50 (55.00-91.00)	69.50 (57.25-88.00)	0.014	0.907
Hematology	Hemoglobin (g/L)	127.50 (106.75-141.25)	130.00 (115.25-143.00)	0.117	0.733
	Red Blood Cell (g/L)	4.32 ± 0.67	4.34 ± 0.68	0.075	0.785
	Platelet Count (10^9^/L)	240.00 (187.50-309.50)	247.00 (199.25-302.50)	0.715	0.398
	Lymphocyte Count (10^9^/L)	1.73 (1.23-2.11)	1.70 (1.31-2.11)	0.154	0.695
	Neutrophil Count (10^9^/L)	4.97 (3.45-7.42)	5.25 (3.70-7.40)	0.543	0.461
	White Blood Cell (10^9^/L)	7.28 (5.44-9.17)	7.35 (5.91-8.73)	0.389	0.533
Other Biomarkers	Uric Acid (μmol/L)	6.51 (4.99-8.12)	6.00 (4.80-7.40)	2.493	0.114
	Procalcitonin (ng/ml)	0.23 (0.19-0.29)	0.23 (0.20-0.28)	0.714	0.398

Mann-Whitney U/t-test for continuous variables; Chi-square/Fisher’s exact test for categorical variables. Significance: P < 0.01.

BP, blood pressure; LDL, low-density lipoprotein; HDL, high-density lipoprotein.

### Selection of predictive factors

**Figure 3:**
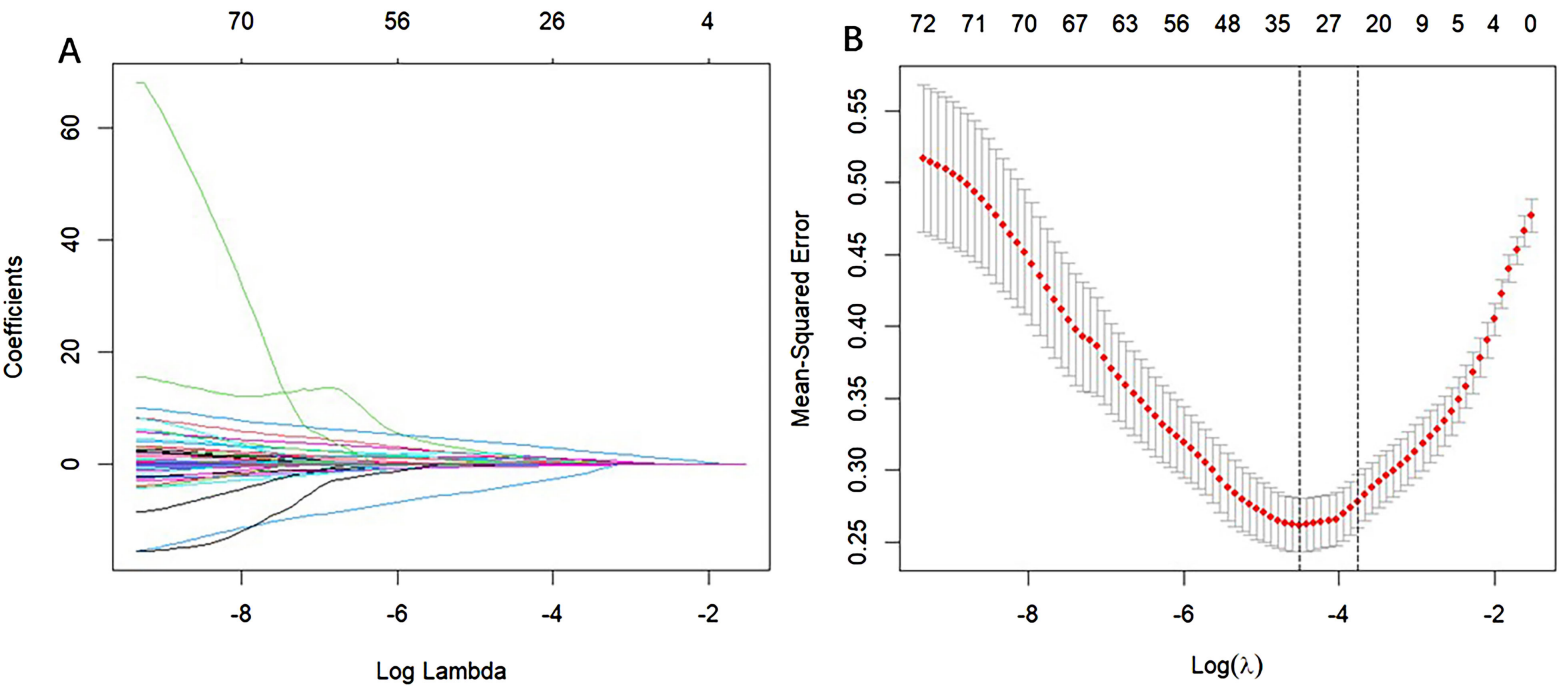
Selection of CHD risk factors using the LASSO regression method. **(A)** In the LASSO model, the penalty parameter (λ) was selected through 1000-fold cross-validation using the minimum criterion. The AUC curve is plotted against log(λ). A dashed line indicates the optimal value determined by both the minimum criterion and the one standard error (1-SE) criterion. Based on the 1000-fold cross-validation, a λ value of 0.05130 was selected, with log(λ) equal to 24.709 (1-SE criterion). **(B)** Distribution of LASSO coefficients for 24 texture features. The coefficient distribution plot is generated against the log(λ) sequence. A vertical line at the value selected from the cross-validation highlights the 9 non-zero coefficients.

The results of univariate regression analysis revealed that several variables were significantly associated with patient grouping (*p* < 0.05). Specifically, the odds ratio (OR) for age was 1.08 (*p* < 0.001), indicating that increasing age is strongly associated with a higher risk of osteoporosis. In terms of gender, the OR for males was 2.05 (*p* = 0.004), suggesting that men are more susceptible to osteoporosis compared to women. Additionally, the OR for alcohol consumption was 2.45 (*p* = 0.015), while the OR for smoking was 1.59 (*p* = 0.138), which did not reach statistical significance. Regarding lipid profiles, the OR for low-density lipoprotein (LDL) was 0.63 (p = 0.001), suggesting a protective effect. Total cholesterol (TC) and triglycerides (TG) showed near-significant effects (p = 0.053). C-reactive protein (CRP) exhibited an OR of 1.01 (p = 0.006), reflecting its association with the inflammatory response. Notably, the OR for red blood cell count (RBC) was 0.28 (p < 0.001), indicating that a lower RBC count is linked to an increased risk of osteoporosis. Multivariate regression analysis further confirmed that age (OR = 1.08, p < 0.001), gender (OR = 2.05, p = 0.004), and RBC count (OR = 0.28, p < 0.001) significantly influence the occurrence of osteoporosis. However, other variables such as alcohol consumption, smoking, and LDL did not show significant associations (p > 0.05). Details are presented in **Table 3** (Full candidate variables are listed in **Supplementary Table S3**). LASSO regression analysis reduced the 24 features to 9 potential predictive factors with non-zero coefficients, which were ultimately included in the LASSO-logistic regression model (**Figure 3** **).** In multivariate analysis, both forward selection and backward elimination methods yielded consistent results. Using forward selection, factors such as age (OR = 1.06, p < 0.001), history of fractures (OR = 85.26, p < 0.001), and alcohol consumption (OR = 12.45, p < 0.001) were significantly associated with osteoporosis, with fracture history having a particularly strong influence on the risk of coronary heart disease (CHD). Backward elimination also validated the impact of total cholesterol (OR = 0.72, p = 0.028) and other variables. Ultimately, the forward selection method was chosen to construct the ROC curve for evaluating the predictive performance of the model due to its superior Akaike Information Criterion (AIC) and fewer independent variables.

**Table 3:** Final multivariable logistic regression model for osteoporosis risk prediction (forward selection).

Characteristics	β	Se	OR	95%CI	Z	*P
Age, years	0.063	0.015	1.06	1.03-1.1	4.115	0
Fracture	4.446	1.134	85.26	9.24-787.11	3.922	0
Packed Cell Volume(%)	-0.135	0.039	0.87	0.81-0.94	-3.485	0
Cl(mmol/L)	-0.13	0.046	0.88	0.8-0.96	-2.835	0.005
Drinking	2.521	0.587	12.45	3.94-39.33	4.297	0
Lymphocyte. Count(%)	-0.581	0.249	0.56	0.34-0.91	-2.334	0.02
Sex	1.186	0.439	3.27	1.38-7.74	2.699	0.007
Totalcholesterol(mmol/L)	-0.286	0.15	0.75	0.56-1.01	-1.916	0.055
Rheumatoid factors(IU/ml)	0.011	0.007	1.01	1-1.02	1.44	0.15
K(mmol/L)	0.625	0.37	1.87	0.9-3.86	1.69	0.091
Alanine Aminotransferase (U/L)	-0.016	0.009	0.98	0.97-1	-1.661	0.097

coefficients (β) standard errors (SE), Odds ratio (OR), P-values were derived from linear regression, Significance level based on Z value *P < 0.05,95% confidence interval for OR(CI).

### Construction of the nomogram prediction model

**Figure 4:**
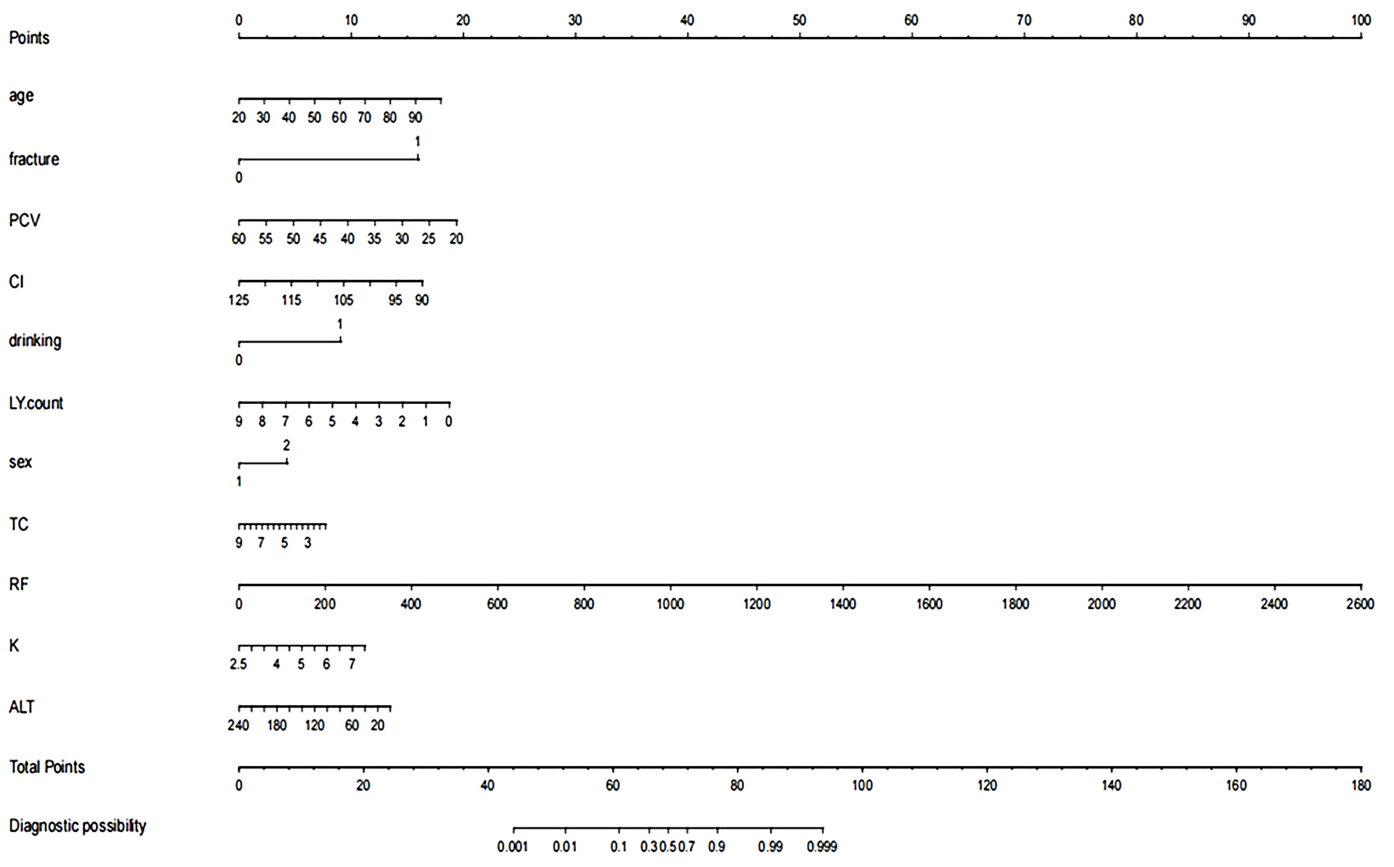
Nomogram of the prediction model. The variables in the figure include age, history of fractures, packed cell volume (PCV), cardiac index (CI), alcohol consumption, lymphocyte count (LY count), gender, total cholesterol (TC), rheumatoid factor (RF), serum potassium (K), and alanine aminotransferase (ALT). Each variable is assigned a specific score (Points) across its value range. The total score is summed and located on the “Total Points” scale below, which is then used to determine the corresponding diagnostic probability on the “Diagnostic Probability” scale at the bottom.

A nomogram prediction model was developed based on the results of the LASSO-logistic regression; refer to **Figure 4** for details.

### Model evaluation

**Figure 5:**
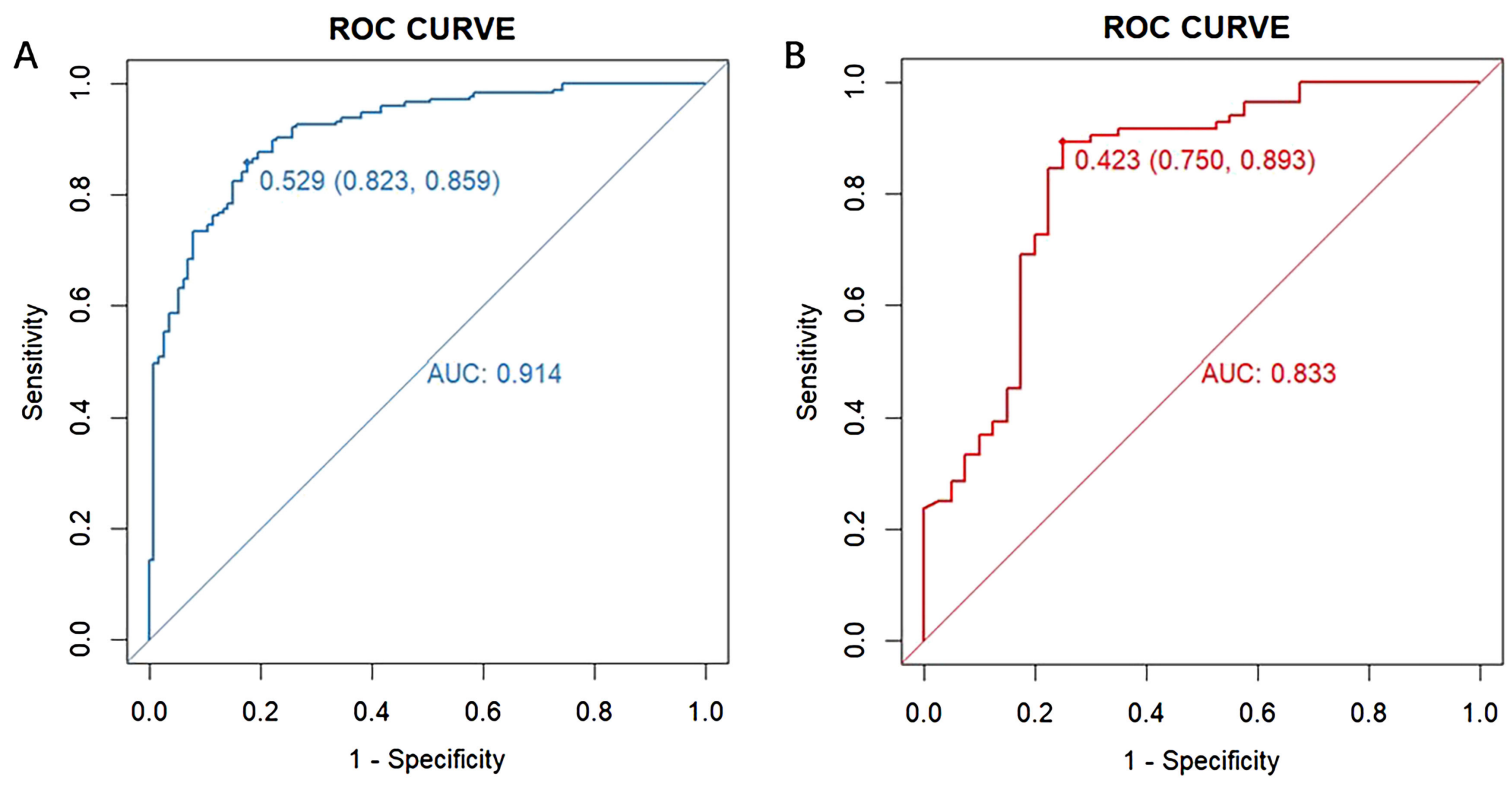
Receiver operating characteristic (ROC) curve for evaluating the diagnostic performance of the model in the training **(A)** and validation **(B)** sets. The left graph displays the ROC curve for the training set (blue curve), and the right graph shows the ROC curve for the validation set (red curve). The horizontal axis represents “1 - Specificity” (false positive rate), and the vertical axis represents “Sensitivity” (true positive rate). The area under each curve (AUC) indicates the model’s ability to distinguish between outcomes, with a value closer to 1 reflecting better model performance.

**Figure 6:**
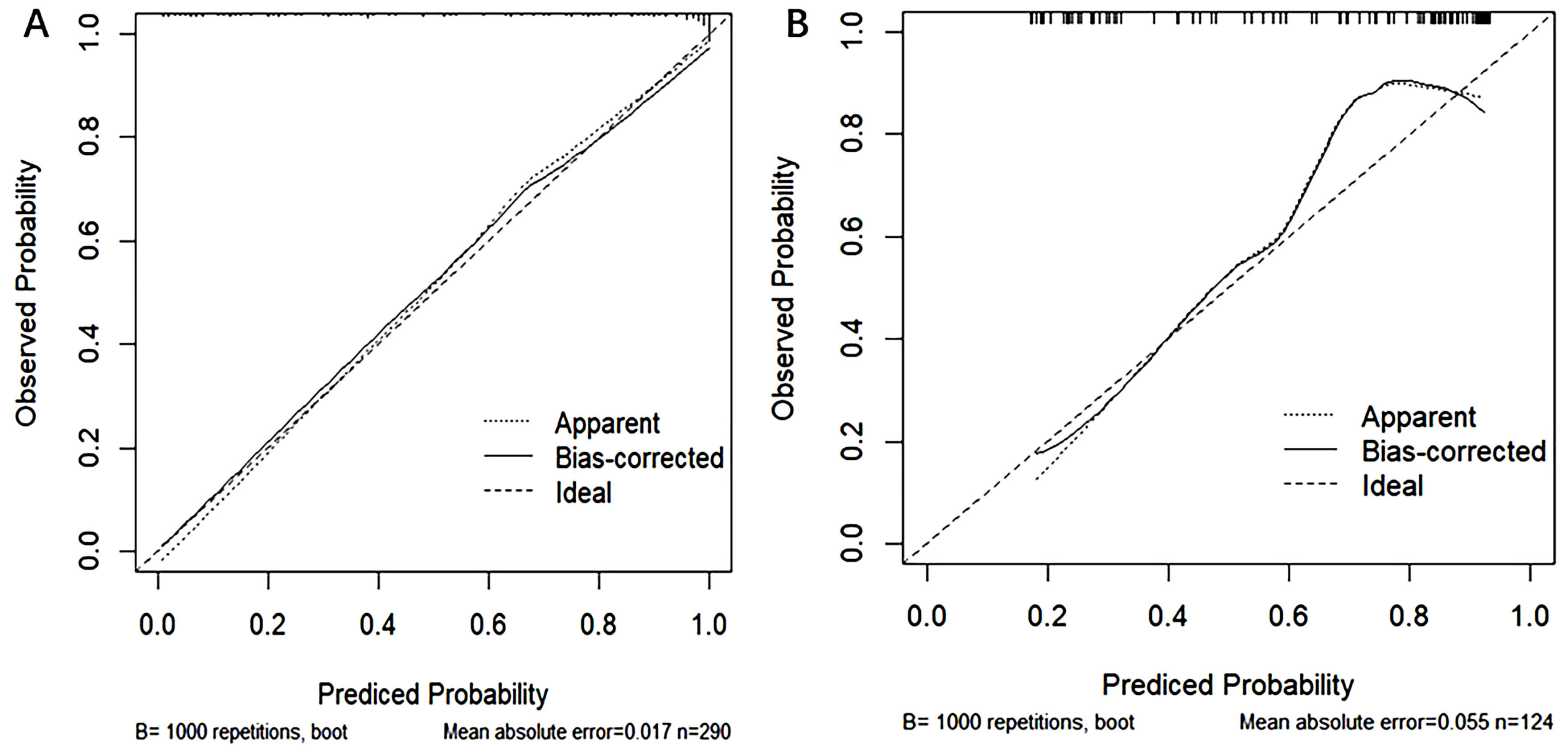
Calibration curves for the training **(A)** and validation **(B)** sets, used to evaluate the consistency between model predicted probabilities and actual observed probabilities. The horizontal axis represents the model-predicted probabilities, while the vertical axis represents the observed probabilities. The dashed line represents the ideal prediction (Ideal), the dotted line shows apparent predictions (Apparent), and the solid line represents predictions after bias correction (Bias-corrected). The left graph illustrates the calibration curve for the training set, while the right graph shows the curve for the validation set.

**Figure 7:**
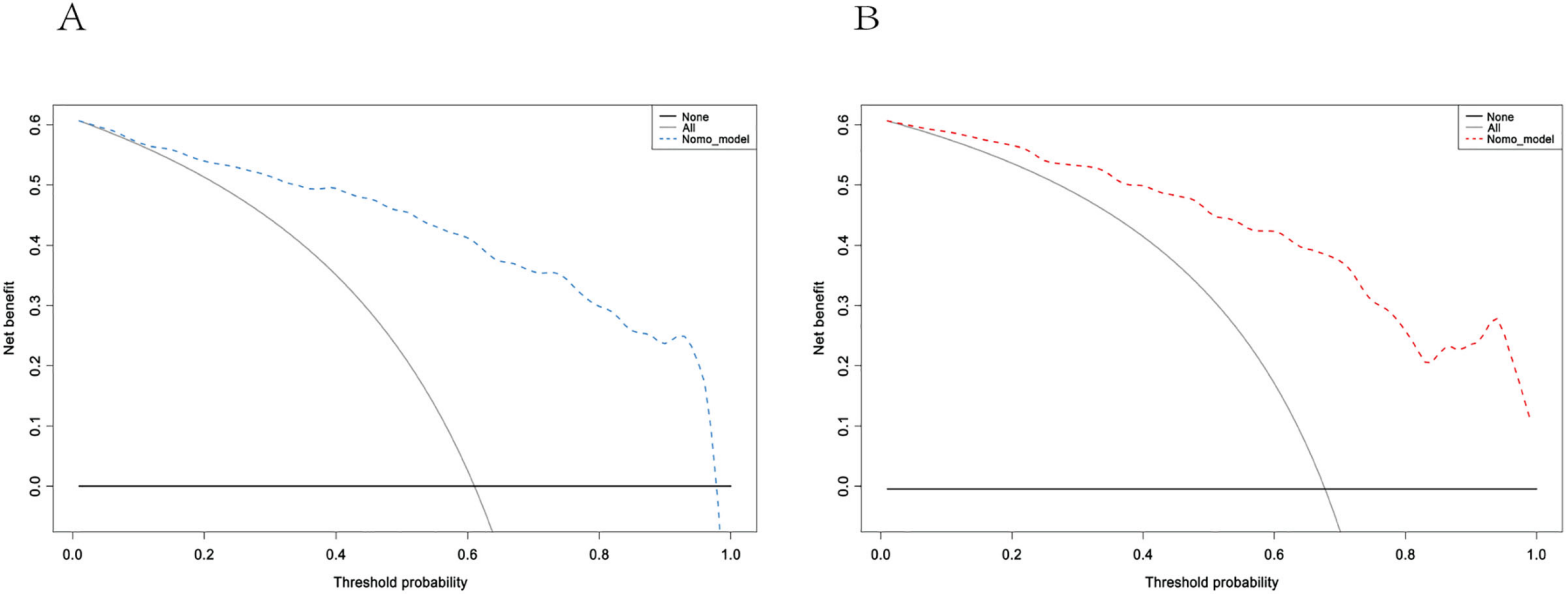
Decision curve Analysis for the training **(A)** and validation **(B)** sets. These curves assess the net benefit of the model across different high-risk thresholds. The left graph shows the blue model curve, while the right graph shows the red model curve. The curve labeled “Model” represents the net benefit of the model, “All” assumes all patients receive intervention, and “None” assumes no intervention for any patients.

The performance of the model was evaluated using ROC curve analysis, showing an AUC of 0.914 for the training set (left graph) and 0.833 for the validation set(right graph) (**Figure 5**). The Hosmer-Lemeshow test results revealed X-squared values of 24.855 (*p* = 0.001645) for the training set and 110.12 (*p* < 2.2e-16) for the validation set, indicating a significant difference in model fit between the two sets. The calibration curves showed good consistency between predicted probabilities and observed outcomes in the training set, with a mean absolute error of 0.017. In the validation set, some bias was observed at higher predicted probabilities, with a mean absolute error of 0.055, suggesting that the model demonstrates good calibration overall (**Figure 6**). Additionally, decision curve analysis (DCA) indicated that the model in the left graph provided greater net benefits for high-risk thresholds ranging from 0 to 0.6, while the model in the right graph showed good net benefits within the 0 to 0.5 range (**Figure 7**). These findings further support the clinical utility and predictive accuracy of the model.

## Discussion

In this study, we developed and validated a machine learning model to predict osteoporosis in patients with low back pain. The model demonstrated robust predictive performance, achieving an AUC of 0.914 in the training set and 0.833 in the validation set, surpassing the accuracy of traditional risk scoring systems. This model effectively estimates the probability of osteoporosis in individuals with low back pain, offering significant potential for clinical applications, particularly in risk assessment and personalized osteoporosis prevention and management. Its implementation could provide valuable support for addressing osteoporosis in remote ethnic minority regions of China, where healthcare resources may be limited.

While most existing osteoporosis prediction models primarily rely on traditional clinical variables, such as age, sex, bone density, and BMI, our study distinguishes itself by incorporating a more comprehensive dataset. Specifically, our model integrates a wide range of clinical hematological and biochemical markers, including serological indicators such as hemoglobin levels, white blood cell count, lymphocyte count, platelet count, triglycerides, and LDL cholesterol. This approach enhances the predictive capability of the model and provides a more holistic assessment of osteoporosis risk.

Among the factors explored, low hemoglobin levels may be linked to chronic low-grade inflammation, oxidative stress, or disruptions in bone metabolism, all of which are potential mechanisms contributing to the development of osteoporosis. Additionally, evidence suggests that very low cholesterol levels, particularly low-density lipoprotein cholesterol (LDL-C), may be associated with decreased bone mineral density and an elevated risk of fractures. This relationship may be mediated by the impact of low cholesterol on estrogen synthesis, which could further exacerbate osteoporosis risk.

Platelets (30) contribute to bone metabolism through multiple mechanisms, including the secretion of cytokines, modulation of osteoclast activity, and involvement in immune responses (31). Furthermore, aging is associated with a decline in osteoblast activity, an increase in osteoclast activity, and enhanced bone resorption. Concurrently, aging reduces intestinal calcium absorption and impairs the synthesis of vitamin D, both of which exacerbate the progression of osteoporosis.

Serum potassium ions exhibit a protective effect on bone health, whereas chloride ions may indirectly influence osteoporosis by modulating calcium excretion (32). In patients with rheumatoid arthritis, the inflammatory response triggers the excessive secretion of cytokines, such as tumor necrosis factor-alpha (TNF-α) and interleukin-6 (IL-6). These cytokines not only perpetuate inflammation but also promote accelerated bone resorption, further contributing to bone loss.

In addition, the activation of the immune system promotes bone resorption through the action of specific immune cells and molecular mechanisms (33). Moreover, the decline in bone density is further exacerbated by long-term medication use (34) and insufficient physical activity (35) in patients with rheumatoid arthritis (RA), highlighting how RA history may trigger or accelerate the progression of osteoporosis (36). Alcohol consumption also plays a significant role in osteoporosis pathogenesis by inhibiting bone formation, increasing bone resorption, disrupting calcium metabolism, and reducing vitamin D activity (37). Notably, a substantial proportion of the population in this region has a history of long-term alcohol consumption, which may further contribute to the burden of osteoporosis.

Lymphocytes contribute to osteoclastogenesis (38) by secreting RANKL (Receptor Activating Factor Ligand) and various cytokines, including TNF-α, IL-6, and IL-17. These cytokines play key roles in regulating bone resorption and formation. Elevated levels of these cytokines have been implicated in promoting excessive bone resorption, reducing bone density, and increasing the risk of osteoporosis. In summary, we selected the aforementioned relevant variables, with a particular focus on exploring how biological mechanisms, such as bone metabolism and the inflammatory response, influence the development of osteoporosis. This approach provided a more robust biological explanation for the variables included in the model. To enhance prediction accuracy and address limitations such as overfitting and insufficient data, we applied LASSO regression and logistic regression to generate calibration plots. The ability to predict osteoporosis risk using simple blood biochemical markers is especially valuable for remote populations with limited access to more precise screening methods.

In this study, we propose column-line plots as a highly accurate and reliable tool for predicting outcomes. These plots are user-friendly and incorporate 11 comprehensive and easily accessible variables, including patient history, age, and blood biomarkers. The model demonstrated consistency indices of 0.914 and 0.833 in the training and validation cohorts, respectively. The calibration curves further confirmed the strong alignment between predicted and actual outcomes. In the osteoporosis risk assessment column chart, multivariate analysis using a bicategorical Logit model for positive selection revealed that age, fracture history, rheumatoid arthritis history, platelet count, serum potassium (K) and chloride (Cl) ions, alcohol consumption history, lymphocyte count, total cholesterol, and serum gamma-glutamyltransferase levels were all strong predictors of osteoporosis risk in patients with lower back pain. In situations where effective bone mineral density (BMD) testing methods are unavailable or patients are unwilling to accept the associated side effects, the likelihood of developing osteoporosis can be predicted by simply gathering information on the patient’s general condition and history. Additionally, completing blood tests for counts, electrolytes (K/Cl), lipids, and serum γ-glutamyl transferase can provide an accurate prediction of osteoporosis incidence, helping to guide interventions and preventive measures to minimize bone-related incidents. Deviation of the calibration curve from the ideal value at high predictive probabilities typically indicates that the model may be overconfident. This issue could stem from an imbalance in the categorical data used for model training or from the model’s reduced generalization ability due to overfitting, particularly when the feature space is more complex. Additionally, these biases may arise from the selection of model features; for instance, weaker associations with osteoporosis may cause biased predictions in high-probability intervals. However, the practical generalizability of the model faces challenges, and further validation across different populations and clinical settings is necessary, especially with multicenter data, to assess its performance on diverse datasets.

Nevertheless, it should be noted that the findings of this study were derived from a single-center cohort within a specific geographical region, potentially limiting their generalizability to broader populations or diverse healthcare settings. The absence of external validation through multicenter datasets or independent cohorts may further constrain the reliability and extrapolation of our results across different demographic groups or regional contexts. Furthermore, it is important to acknowledge that numerous clinically significant risk factors for osteoporosis may not have been adequately accounted for in this investigation, primarily due to inherent constraints in data acquisition and study design. While the developed model demonstrates promising potential for facilitating personalized treatment strategies, thereby potentially mitigating fracture risk and enhancing patient outcomes, several limitations warrant consideration. Most notably, the model’s predictive performance, though robust, is constrained by the modest sample size and the single-center origin of the dataset. To enhance the model’s clinical applicability and external validity, future studies should prioritize multicenter validation with larger, more diverse cohorts. Additionally, the collection and incorporation of comprehensive clinical data from varied healthcare settings across different geographical regions will be essential to rigorously evaluate the model’s generalizability and ensure its reliability across heterogeneous populations.

Future research directions should focus on three key aspects to enhance the clinical utility of the model: First, optimization and refinement of the algorithm should be pursued, incorporating advanced model interpretation methodologies to improve transparency and provide clinicians with more robust decision-making support. Second, integration with existing healthcare infrastructure, particularly electronic health record systems and clinical decision support systems, should be implemented to enable real-time prediction capabilities and facilitate rapid clinical decision-making. Third, the model’s architecture should be expanded to incorporate additional predictive variables, including region-specific lifestyle factors, genetic predisposition markers, and comprehensive metabolic indicators, thereby enhancing its accuracy and broadening its applicability. These strategic enhancements will be crucial for validating the model’s performance across diverse populations and clinical settings.

The novel predictive model for osteoporosis risk assessment, specifically developed for ethnic minority regions, demonstrates robust predictive performance through the innovative integration of clinical features and biomarker profiles. This model represents a significant advancement in osteoporosis screening, particularly for resource-limited border areas of China, with special relevance to the Wenshan Zhuang and Miao Autonomous Prefecture in Yunnan Province. By providing a reliable screening tool that circumvents the need for specialized equipment, this model addresses critical healthcare disparities in underserved regions. Furthermore, the proposed predictive framework establishes a new paradigm for early detection and timely intervention of osteoporosis, potentially transforming current screening practices in resource-constrained settings.

## Data Availability

The original contributions presented in the study are included in the article/**Supplementary Material**. Further inquiries can be directed to the corresponding authors.

## References

[1] Ferreira ML De Luca K Haile LM Steinmetz JD Culbreth GT Cross M. Global, regional, and national burden of low back pain, 1990–2020, its attributable risk factors, and projections to 2050: a systematic analysis of the Global Burden of Disease Study 2021. Lancet Rheumatol. (2023) 5:e316–29. doi: 10.1016/S2665-9913(23)00098-X PMC10234592 37273833

[2] Pergolizzi JV LeQuang JA. Rehabilitation for low back pain: A narrative review for managing pain and improving function in acute and chronic conditions. Pain Ther. (2020) 9:83–96. doi: 10.1007/s40122-020-00149-5 32006236 PMC7203283

[3] Wang J. Transition from acute to chronic pain: evaluating risk for chronic postsurgical pain. Pain Phys. (2019) 5:479–88. doi: 10.36076/ppj/2019.22.479 31561647

[4] Clark EM Gooberman-Hill R Peters TJ. Using self-reports of pain and other variables to distinguish between older women with back pain due to vertebral fractures and those with back pain due to degenerative changes. Osteoporos Int. (2016) 27:1459–67. doi: 10.1007/s00198-015-3397-2 PMC4791465 26564228

[5] Bours SPG Van Den Bergh JPW Van Geel TACM Geusens PPMM. Secondary osteoporosis and metabolic bone disease in patients 50 years and older with osteoporosis or with a recent clinical fracture: a clinical perspective. Curr Opin Rheumatol. (2014) 26:430–9. doi: 10.1097/BOR.0000000000000074 24841229

[6] Rousière M. De l’importance de prendre en charge l’ostéoporose. La Presse Médicale. (2011) 40:900–9. doi: 10.1016/j.lpm.2011.02.025 21493035

[7] Dhainaut A Hoff M Syversen U Haugeberg G. Technologies for assessment of bone reflecting bone strength and bone mineral density in elderly women: an update. Womens Health (Lond Engl). (2016) 12:209–16. doi: 10.2217/whe.15.94 PMC5375053 26900798

[8] Dal Canto E Ceriello A Rydén L Ferrini M Hansen TB Schnell O. Diabetes as a cardiovascular risk factor: An overview of global trends of macro and micro vascular complications. Eur J Preventive Cardiol. (2019) 26:25–32. doi: 10.1177/2047487319878371 31722562

[9] Leon BM. Diabetes and cardiovascular disease: Epidemiology, biological mechanisms, treatment recommendations and future research. WJD. (2015) 6:1246. doi: 10.4239/wjd.v6.i13.1246 26468341 PMC4600176

[10] Regassa LD Tola A Ayele Y. Prevalence of cardiovascular disease and associated factors among type 2 diabetes patients in selected hospitals of Harari Region, Eastern Ethiopia. Front Public Health. (2021) 8:532719. doi: 10.3389/fpubh.2020.532719 33614562 PMC7892600

[11] Kalra S Aggarwal S Khandelwal D. Thyroid dysfunction and type 2 diabetes mellitus: screening strategies and implications for management. Diabetes Ther. (2019) 10:2035–44. doi: 10.1007/s13300-019-00700-4 PMC6848627 31583645

[12] Li L Liu S Yu J. Autoimmune thyroid disease and type 1 diabetes mellitus: same pathogenesis; new perspective? Ther Adv Endocrinol. (2020) 11:2042018820958329. doi: 10.1177/2042018820958329 PMC7493255 32973994

[13] Yasuda S Inoue I Shimada A. Neurofibromatosis type 1 with concurrent multiple endocrine disorders: adenomatous goiter, primary hyperparathyroidism, and acromegaly. Intern Med. (2021) 60:2451–9. doi: 10.2169/internalmedicine.4981-20 PMC8381186 34334593

[14] Liu M Zhang Y Cheng X Lu Y Li N Gong Y. The effect of age on the changes in bone mineral density and osteoporosis detection rates in Han Chinese men over the age of 50. Aging Male. (2014) 17:166–73. doi: 10.3109/13685538.2014.940308 25027466

[15] Xu Z Zhou Y Wu X Li H Bian W. Bone health and awareness of osteoporosis in women aged 40 to 60 years in Jiaxing City, China. Medicine. (2024) 103:e38073. doi: 10.1097/MD.0000000000038073 38728513 PMC11081584

[16] Su B Li D Xie J Wang Y Wu X Li J. Chronic disease in China: geographic and socioeconomic determinants among persons aged 60 and older. J Am Med Directors Assoc. (2023) 24:206–212.e5. doi: 10.1016/j.jamda.2022.10.002 36370750

[17] De Jong MR van der Elst M Hartholt KA. Drug-related falls in older patients: implicated drugs, consequences, and possible prevention strategies. Ther Adv Drug Saf. (2013) 4:147–54. doi: 10.1177/2042098613486829 PMC4125318 25114778

[18] Petryla G Uvarovas V Bobina R Kurtinaitis J Khan SA Versocki A. The one-year mortality rate in elderly patients with osteoporotic fractures of the pelvis. Arch Osteoporos. (2020) 15:15. doi: 10.1007/s11657-020-0689-8 32078053

[19] Rinonapoli G Ruggiero C Meccariello L Bisaccia M Ceccarini P Caraffa A. Osteoporosis in men: A review of an underestimated bone condition. IJMS. (2021) 22:2105. doi: 10.3390/ijms22042105 33672656 PMC7924179

[20] Leslie WD Crandall CJ. Population-based osteoporosis primary prevention and screening for quality of care in osteoporosis, current osteoporosis reports. Curr Osteoporos Rep. (2019) 17:483–90. doi: 10.1007/s11914-019-00542-w 31673933

[21] Dimai HP. Use of dual-energy X-ray absorptiometry (DXA) for diagnosis and fracture risk assessment; WHO-criteria, T- and Z-score, and reference databases. Bone. (2017) 104:39–43. doi: 10.1016/j.bone.2016.12.016 28041872

[22] Jones AC Woldemikael D Fisher T Hobbs GR Prud’homme BJ Bal GK. Repeated computed tomographic scans in transferred trauma patients: Indications, costs, and radiation exposure. J Trauma Acute Care Surg. (2012) 73:1564–9. doi: 10.1097/TA.0b013e31826fc85f 23147177

[23] Lin X Xiong D Peng YQ Sheng ZF Wu XY Wu XP. Epidemiology and management of osteoporosis in the People's Republic of China: current perspectives.. Clin Interv Aging. (2015) 10:1017–33. doi: 10.2147/CIA.S54613 PMC4485798 26150706

[24] Cauley JA Barbour KE Harrison SL Cloonan YK Danielson ME Ensrud KE. Inflammatory markers and the risk of hip and vertebral fractures in men: the osteoporotic fractures in men (MrOS). J Bone Mineral Res. (2016) 31:2129–38. doi: 10.1002/jbmr.2905 PMC5240475 27371811

[25] Li H Zhang X Zhang Q Zhang Q Zhu X Xie T. The relationship between the monocyte-to-lymphocyte ratio and osteoporosis in postmenopausal females with T2DM: A retrospective study in Chinese population. Front Endocrinol. (2023) 14:1112534. doi: 10.3389/fendo.2023.1112534 PMC9987332 36891058

[26] Li B Shang Q Jing R Qin W Liu H Dai X. Correlation analysis between hemoglobin and osteoporosis fractures in elderly patients. Chin J Osteopros. (2024) 30:27–31. doi: 10.3969/j.issn.1006-7108.2024.01.006

[27] Lu Z Lyu L Han Y Ma Z Kong H. Association between serum uric acid/high-density lipoprotein cholesterol ratio and bone mineral density in elderly patients with type 2 diabetes mellitus. Lab Med Clinic. (2025) 22(03):299–303. Available online at: https://link.cnki.net/urlid/50.1167.r.20250117.1313.004.

[28] Chen Y Sun K Li N Wang C Huang C Li L. Analysis of the correlation between plasma fibrinogen and osteoporosis defined by quantitative computed tomography. J Of Sun Yat⁃Sen University(Medical Sciences). (2025) 46:1672–3554. doi: 10.13471/j.cnki.j.sun.yat-sen.univ(med.sci).2025.0117

[29] Guo Q Yao R Yang H Yang K. Application research progress of red blood cell distribution width in orthopedics. ACM. (2024) 14:474–9. doi: 10.12677/ACM.2024.143726

[30] Chen X Wang Z Duan N Zhu G Schwarz EM Xie C. Osteoblast–osteoclast interactions. Connective Tissue Res. (2018) 59:99–107. doi: 10.1080/03008207.2017.1290085 PMC5612831 28324674

[31] Tjhia CK Odvina CV Rao DS Stover SM Wang X Fyhrie DP. Mechanical property and tissue mineral density differences among severely suppressed bone turnover (SSBT) patients, osteoporotic patients, and normal subjects. Bone. (2011) 49:1279–89. doi: 10.1016/j.bone.2011.09.042 PMC3221814 21958843

[32] Orsini F Crotti C Cincinelli G Di Taranto R Amati A Ferrito M. Bone involvement in rheumatoid arthritis and spondyloartritis: an updated review. Biology. (2023) 12:1320. doi: 10.3390/biology12101320 37887030 PMC10604370

[33] Bugatti S Bogliolo L Manzo A De Stefano L Delvino P Motta F. Impact of anti-citrullinated protein antibodies on progressive systemic bone mineral density loss in patients with early rheumatoid arthritis after two years of treat-to-target. Front Immunol. (2021) 12:701922. doi: 10.3389/fimmu.2021.701922 34194443 PMC8236980

[34] Hagen KB Dagfinrud H Moe RH Østerås N Kjeken I Grotle M. Exercise therapy for bone and muscle health: an overview of systematic reviews. BMC Med. (2012) 10:167. doi: 10.1186/1741-7015-10-167 23253613 PMC3568719

[35] Li M Zhang Z Xia W. Interpretation on the guidelines for the diagnosis and treatment of primary osteoporosis(2022). Med J Peking Union Med Coll Hosp. (2023) 14:1203–7. doi: 10.12290/xhyxzz.2023-0364

[36] Kareem R Botleroo RA Bhandari R Ogeyingbo OD Ahmed R Gyawali M. The impact of rheumatoid arthritis on bone loss: links to osteoporosis and osteopenia. Cureus. (2021) 13:e17519. doi: 10.7759/cureus.17519 34603889 PMC8476196

[37] Zhu D Fang H Yu H Liu P Yang Q Luo P. Alcohol-induced inhibition of bone formation and neovascularization contributes to the failure of fracture healing via the miR-19a-3p/FOXF2 axis. Bone Joint Res. (2022) 11:386–97. doi: 10.1302/2046-3758.116.BJR-2021-0596.R1 PMC9233406 35730670

[38] Man Q Zhang L Zhao Y Liu J Zheng Y Zhao Y. Lymphocyte−derived microparticles stimulate osteoclastogenesis by inducing RANKL in fibroblasts of odontogenic keratocysts. Oncol Rep. (2018) 40:3335–45. doi: 10.3892/or.2018.6708 30272301

