# Supplementary material for: A novel nomogram for predicting osteoporosis with low back pain among the patients in Wenshan Zhuang and Miao Autonomous Prefecture of China
Source: Front Endocrinol (Lausanne). 2025 Jun 5;16:1535163. doi: 10.3389/fendo.2025.1535163 (PMC12176570; doi:10.3389/fendo.2025.1535163)
Supplement: Supplementary file 1 [file Table1.docx]

**Table S1**. Baseline Characteristics of Osteoporosis and Non-Osteoporosis

Groups in Low Back Pain Patients (N=414)

| Characteristic | Osteoporosis. 0 | Osteoporosis. 1 | Statistical Value | P Value |
| --- | --- | --- | --- | --- |
| N | 153 | 261 |  |  |
| Systolic blood pressure (mmHg) | 128.00 (118.00-143.00) | 130.00(118.00-146.00) | 1.285 | 0.257 |
| Diastolic blood pressure (mmHg) | 80.00 (71.00-88.00) | 78.00 (70.00-88.00) | 0.511 | 0.475 |
| Age,years | 62.00 (53.00-73.00) | 75.00 (69.00-82.00) | 82.601 | <0.001 |
| Totalcholesterol(mmol/L) | 5.10 (4.21-6.03) | 4.43 (3.75-5.25) | 21.386 | <0.001 |
| Triglyceride(mmol/L) | 1.44 (1.06-2.44) | 1.37 (1.05-1.87) | 3.061 | 0.08 |
| High Density Lipoprotein(mmol/L) | 1.24 (1.06-1.45) | 1.22 (1.03-1.45) | 0.514 | 0.473 |
| Low Density Lipoprotein (mmol/L) | 2.96 (2.25-3.48) | 2.52 (1.94-3.11) | 18.376 | <0.001 |
| Creative kinase isoenzyme MB(ng/ml) | 19.00 (15.00-23.00) | 16.00 (12.00-21.00) | 10.405 | 0.001 |
| C-reactive protein(mg/L) | 3.80 (1.40-10.60) | 8.60 (3.80-51.60) | 28.424 | <0.001 |
| Ca(mmol/L) | 2.25 (2.18-2.32) | 2.23 (2.15-2.31) | 3.213 | 0.073 |
| procalcitonin(ng/ml) | 0.04 (0.03-0.10) | 0.07 (0.04-0.23) | 15.923 | <0.001 |
| Uric Acid(umol/L) | 329.00 (274.00-400.00) | 308.00 (247.00-410.00) | 2.17 | 0.141 |
| Hemoglobin(g/L) | 139.00 (127.00-149.00) | 123.00 (99.00-137.00) | 49.906 | <0.001 |
| Glucose(mmol/L) | 5.14 (4.79-6.03) | 5.49 (4.89-6.53) | 7.429 | 0.006 |
| Fibrinogen(g/L) | 3.14 (2.70-4.10) | 3.57 (2.76-4.81) | 6.088 | 0.014 |
| D.dimer(mg/L) | 0.59 (0.29-1.94) | 1.11 (0.44-2.91) | 15.287 | <0.001 |
| γ-Glutamyl Transferase(U/L) | 30.00 (22.00-54.00) | 27.00 (17.00-47.00) | 4.083 | 0.043 |
| Alanine Aminotransferase (U/L) | 20.00 (14.00-35.00) | 16.00 (9.00-26.00) | 15.675 | <0.001 |
| Aspartate Aminotransferase (U/L) | 21.00 (18.00-29.00) | 23.00 (18.00-32.00) | 1.31 | 0.252 |
| Albumin(g/L) | 39.90 (37.20-42.10) | 37.80 (35.10-40.60) | 19.133 | <0.001 |
| White Blood Cell (g/L) | 7.31 (5.71-8.84) | 7.37 (5.71-8.88) | 0.237 | 0.626 |
| Red Blood Cell(g/L) | 4.62±0.59 | 4.16±0.66 | 50.143 | <0.001 |
| Packed Cell Volume(%) | 42.20 (38.90-44.70) | 38.30 (33.50-41.70) | 51.261 | <0.001 |
| Creative kinase(ng/ml) | 78.00 (58.00-98.00) | 68.00 (45.00-92.00) | 6.899 | 0.009 |
| Creatinine(umol/L) | 67.00 (56.00-82.00) | 71.00 (58.00-92.00) | 3.665 | 0.056 |
| Alkaline phosphatase(U/L) | 84.00 (71.00-97.00) | 87.00 (73.00-102.00) | 1.82 | 0.177 |
| Urea nitrogen(mmol/L) | 6.10 (5.00-7.40) | 6.20 (4.88-7.70) | 0.153 | 0.696 |
| Mean Corpuscular Volume(fL) | 92.80 (89.00-95.30) | 93.10 (88.80-97.00) | 2.892 | 0.089 |
| Mean Corpuscular Hemoglobin(pg) | 30.50 (29.40-31.40) | 30.70 (29.10-32.00) | 0.825 | 0.364 |
| Mean Corpuscular Hemoglobin Concentration(g/L) | 331.97±8.49 | 330.85±10.27 | 1.307 | 0.254 |
| Lactate Dehydrogenase(U/L) | 213.00 (180.00-257.00) | 221.00 (183.00-270.00) | 2.258 | 0.133 |
| Platelet Count(10×109/L) | 248.00 (207.00-301.00) | 240.00 (191.00-304.00) | 0.332 | 0.564 |
| Total Bilirubin(umol/L) | 9.50 (8.10-13.10) | 9.50 (7.30-14.70) | 0.142 | 0.706 |

| Characteristic | Osteoporosis. 0 | Osteoporosis. 1 | Statistical Value | P Value |
| --- | --- | --- | --- | --- |
| Direct Bilirubin(umol/L) | 3.80 (3.10-5.30) | 4.30 (3.10-6.30) | 2.639 | 0.104 |
| Indirect Bilirubin(umol/L) | 7.20 (5.60-9.10) | 6.60 (4.50-9.00) | 3.073 | 0.08 |
| Total protein(g/L) | 66.28±6.21 | 65.17±6.76 | 2.772 | 0.097 |
| Albumin/Globulin(%) | 1.60 (1.40-1.70) | 1.50 (1.30-1.70) | 10.458 | 0.001 |
| Large platelet ratio(%) | 24.60 (19.70-30.00) | 25.30 (19.50-31.70) | 0.524 | 0.469 |
| Monocyte.percent(%) | 6.40 (5.20-7.60) | 7.00 (5.90-8.50) | 13.432 | <0.001 |
| Monocyte.count(10×109/L) | 0.48 (0.34-0.67) | 0.50 (0.37-0.66) | 1.587 | 0.208 |
| Lymphocyte.percent(%) | 27.50 (17.80-35.40) | 24.10 (10.60-32.20) | 5.068 | 0.024 |
| Lymphocyte.count(10×109/L) | 1.93 (1.55-2.21) | 1.59 (1.17-2.01) | 22.571 | <0.001 |
| Thyroxine(pmol/L) | 99.81 (84.13-117.30) | 95.09 (78.60-118.50) | 0.842 | 0.359 |
| K(mmol/L) | 4.11 (3.76-4.36) | 4.16 (3.83-4.54) | 4.873 | 0.027 |
| Cl(mmol/L) | 107.80 (105.80-109.20) | 106.50 (99.90-108.90) | 16.046 | <0.001 |
| Na(mmol/L) | 141.30 (140.00-142.50) | 141.40 (139.70-142.80) | 0.017 | 0.897 |
| Mg(mmol/L) | 0.89 (0.83-0.95) | 0.89 (0.81-0.95) | 0.085 | 0.771 |
| Mean Platelet Volume(fL) | 9.60 (9.00-10.10) | 9.60 (9.00-10.00) | 0.108 | 0.743 |
| Globulin(g/L) | 26.10 (23.40-29.40) | 27.50 (24.00-31.00) | 3.986 | 0.046 |
| Basophil.percent(%) | 0.50 (0.40-0.60) | 0.50 (0.30-0.70) | 0.272 | 0.602 |
| Basophil.count(10×109/L) | 0.03 (0.02-0.05) | 0.03 (0.02-0.05) | 0.968 | 0.325 |
| Free Thyroxine (pmol/L) | 16.62 (14.88-18.38) | 16.75 (14.96-18.89) | 0.313 | 0.576 |
| Total bile acids (umol/L) | 4.50 (3.20-6.40) | 4.70 (2.90-7.80) | 0.217 | 0.642 |
| Platelet Distribution Width(%) | 16.10 (15.80-16.40) | 16.00 (15.70-16.40) | 3.364 | 0.067 |
| Procalcitonin(%) | 0.23 (0.20-0.28) | 0.23 (0.19-0.29) | 0.573 | 0.449 |
| Immature Granulocyte.percent(%) | 0.20 (0.10-0.60) | 0.30 (0.10-0.60) | 1.194 | 0.275 |
| Immature Granulocyte.count(fL) | 0.01 (0.01-0.05) | 0.02 (0.01-0.05) | 0.883 | 0.347 |
| Neutrophil.percent(%) | 70.10 (59.20-81.20) | 72.80 (64.40-82.40) | 3.617 | 0.057 |
| Neutrophil.count(fL) | 5.05 (3.63-7.48) | 5.25 (3.65-7.40) | 0.085 | 0.771 |
| Rheumatoid factors(IU/ml) | 3.00 (2.00-8.00) | 5.00 (2.00-13.00) | 8.396 | 0.004 |
| dataset | 1.00 (0.00-1.00) | 1.00 (0.00-1.00) | 1.673 | 0.196 |
| sex |  |  | 11.384 | <0.001 |
| 1（male） | 69 (45.10%) | 75 (28.74%) |  |  |
| 2(female) | 84 (54.90%) | 186 (71.26%) |  |  |
| osteoporosis |  |  | 414 | <0.001 |
| 0 | 153 (100.00%) | 0 (0.00%) |  |  |
| 1 | 0 (0.00%) | 261 (100.00%) |  |  |
| nation |  |  | 30.721 | <0.001 |
| 1(Han) | 114 (74.51%) | 195 (74.71%) |  |  |
| 2(Zhuang) | 24 (15.69%) | 40 (15.33%) |  |  |
| 3(Yi) | 0 (0.00%) | 16 (6.13%) |  |  |
| 4(Miao) | 0 (0.00%) | 6 (2.30%) |  |  |
| 5(Yao) | 6 (3.92%) | 1 (0.38%) |  |  |
| 6(Hui) | 0 (0.00%) | 1 (0.38%) |  |  |

| Statistical | | | | |
| --- | --- | --- | --- | --- |
| Characteristic | Osteoporosis. 0 | Osteoporosis. 1 | Value | P Value |
| 7(Tu Jia) | 6 (3.92%) | 2 (0.77%) |  |  |
| 13(MengGu) | 1 (0.65%) | 0 (0.00%) |  |  |
| 19(Bai)  History | 1 (0.65%) | 0 (0.00%) |  |  |
| smoking |  |  | 0.659 | 0.417 |
| 0 | 128 (83.66%) | 210 (80.46%) |  |  |
| 1 | 25 (16.34%) | 51 (19.54%) |  |  |
| drinking |  |  | 3.189 | 0.074 |
| 0 | 137 (89.54%) | 217 (83.14%) |  |  |
| 1 | 16 (10.46%) | 44 (16.86%) |  |  |
| hypertension |  |  | 1.892 | 0.169 |
| 0 | 95 (62.09%) | 144 (55.17%) |  |  |
| 1 | 58 (37.91%) | 117 (44.83%) |  |  |
| cerebral.infarction |  |  | 4.273 | 0.039 |
| 0 | 151 (98.69%) | 247 (94.64%) |  |  |
| 1 | 2 (1.31%) | 14 (5.36%) |  |  |
| encephalalatrophy |  |  | 3.108 | 0.078 |
| 0 | 147 (96.08%) | 239 (91.57%) |  |  |
| 1 | 6 (3.92%) | 22 (8.43%) |  |  |
| Geriatric.brain.changes |  |  | 1.71 | 0.191 |
| 0 | 152 (99.35%) | 261 (100.00%) |  |  |
| 1 | 1 (0.65%) | 0 (0.00%) |  |  |
| pneumonia |  |  | 0.129 | 0.72 |
| 0 | 141 (92.16%) | 243 (93.10%) |  |  |
| 1 | 12 (7.84%) | 18 (6.90%) |  |  |
| pnlmonary.nodule |  |  | 3.021 | 0.082 |
| 0 | 142 (92.81%) | 228 (87.36%) |  |  |
| 1 | 11 (7.19%) | 33 (12.64%) |  |  |
| hyperosteogeny |  |  | 0.034 | 0.853 |
| 0 | 151 (98.69%) | 257 (98.47%) |  |  |
| 1 | 2 (1.31%) | 4 (1.53%) |  |  |
| atherosclerosis |  |  | 2.638 | 0.104 |
| 0 | 152 (99.35%) | 253 (96.93%) |  |  |
| 1 | 1 (0.65%) | 8 (3.07%) |  |  |
| rheumatoid.arthritis |  |  | 4.933 | 0.026 |
| 0 | 152 (99.35%) | 249 (95.40%) |  |  |
| 1 | 1 (0.65%) | 12 (4.60%) |  |  |
| fracture |  |  | 48.792 | <0.001 |
| 0 | 151 (98.69%) | 185 (70.88%) |  |  |
| 1 | 2 (1.31%) | 76 (29.12%) |  |  |

Continuous variables presented as mean (SD), median (IQR) based on distributionor n (%).

Percentages might not total 100 because of rounding. Continuous variables were compared using Student's t-test (normally distributed data) or Mann-Whitney U test (non-normally distributed data); categorical variables were analyzed by Chi-square test or Fisher's exact test. P<0.01 vs. control group. 0 = non-osteoporosis, 1 = osteoporosis. 0=negative, 1=positive
