# Supplementary material for: A novel nomogram for predicting osteoporosis with low back pain among the patients in Wenshan Zhuang and Miao Autonomous Prefecture of China
Source: Front Endocrinol (Lausanne). 2025 Jun 5;16:1535163. doi: 10.3389/fendo.2025.1535163 (PMC12176570; doi:10.3389/fendo.2025.1535163)
Supplement: Supplementary file 2 [file Table2.docx]

**Table S2．**summarizes the baseline characteristics of confirmed and unconfirmed osteoporosis patients in the training and validation cohorts.

| Characteristic | Validation | Train | Statistical Value | P Value |
| --- | --- | --- | --- | --- |
| N | 124 | 290 |  |  |
| Systolic blood pressure(mmHg) | 129.00(119.00-144.50) | 130.00(117.00-146.00) | 0.01 | 0.92 |
| Diastolic blood pressure(mmHg) | 76.50(67.75-86.00) | 80.00 (70.00-89.00) | 4.803 | 0.028 |
| age,years | 73.00(63.75-80.25) | 72.00 (60.00-79.00) | 0.271 | 0.603 |
| Totalcholesterol  (mmol/L) | 4.77 (3.92-5.66) | 4.62 (3.91-5.51) | 0.706 | 0.401 |
| Triglyceride  (mmol/L) | 1.52 (1.10-2.27) | 1.35 (1.03-2.06) | 3.774 | 0.052 |
| High Density Lipoprotein (mmol/L) | 1.22 (1.04-1.41) | 1.24 (1.03-1.47) | 0.205 | 0.651 |
| Low Density Lipoprotein (mmol/L) | 2.73 (2.08-3.26) | 2.68 (2.07-3.32) | <0.001 | 0.975 |
| Creative kinaseisoenzyme MB(ng/ml) | 17.00(13.00-20.00) | 17.00 (13.00-22.00) | 0.363 | 0.547 |
| C-reactive protein(mg/L) | 7.00 (2.58-34.20) | 6.20 (2.24-32.52) | 0.586 | 0.444 |
| Ca(mmol/L) | 2.25 (2.16-2.31) | 2.24 (2.16-2.32) | 0.068 | 0.794 |
| procalcitonin(ng/ml) | 0.06 (0.04-0.22) | 0.05 (0.04-0.17) | 0.204 | 0.651 |
| Uric Acid (umol/L) | 308.00 (251.75-378.50) | 320.50 (266.00-411.75) | 1.87 | 0.171 |
| Hemoglobin (g/L) | 127.50 (106.75-141.25) | 130.00 (115.25-143.00) | 0.117 | 0.733 |
| Glucose (mmol/L) | 5.34 (4.86-6.39) | 5.36 (4.81-6.33) | 0.338 | 0.561 |
| Fibrinogen(g/L) | 3.46 (2.80-4.62) | 3.33 (2.68-4.45) | 0.834 | 0.361 |
| D.dimer(mg/L) | 1.00 (0.44-2.84) | 0.84 (0.35-2.37) | 1.646 | 0.2 |
| γ-Glutamyl Transferase (U/L) | 29.00 (18.00-49.50) | 28.00 (19.00-49.75) | 0.016 | 0.901 |
| Alanine Aminotransferase (U/L) | 18.00 (11.00-29.25) | 17.00 (11.00-28.75) | 0.159 | 0.69 |
| Aspartate Aminotransferase (U/L) | 23.00 (18.00-31.00) | 22.00 (18.00-30.00) | 0.046 | 0.83 |
| Albumin (g/L) | 39.15 (36.10-41.20) | 38.80 (35.73-41.35) | 0.519 | 0.471 |
| White Blood Cell (g/L) | 7.28 (5.44-9.17) | 7.35 (5.91-8.73) | 0.389 | 0.533 |
| Red Blood Cell (g/L) | 4.32±0.67 | 4.34±0.68 | 0.075 | 0.785 |
| Creative kinase(ng/ml) | 73.00 (50.50-95.25) | 71.00 (48.00-92.75) | 0.014 | 0.906 |
| Creatinine(umol/L) | 68.50 (55.00-91.00) | 69.50 (57.25-88.00) | 0.014 | 0.907 |
| Alkaline phosphatase(U/L) | 86.00 (73.75-98.00) | 85.50 (71.00-99.75) | 0.039 | 0.843 |
| Urea nitrogen(mmol/L) | 6.51 (4.99-8.12) | 6.00 (4.80-7.40) | 2.493 | 0.114 |
| Mean Corpuscular Volume (fL) | 92.70 (88.88-95.43) | 93.10 (88.93-96.10) | 0.016 | 0.898 |
| Mean Corpuscular Hemoglobin (pg) | 30.55 (29.30-31.72) | 30.60 (29.20-31.80) | 0.099 | 0.753 |
| Mean Corpuscular Hemoglobin Concentration (g/L) | 331.81±9.52 | 331.03±9.72 | 0.571 | 0.45 |
| Lactate Dehydrogenase (U/L) | 219.50 (181.00-260.00) | 217.50 (182.25-267.75) | 0.008 | 0.93 |
| Platelet Count (10×10^9^/L) | 240.00 (187.50-309.50) | 247.00 (199.25-302.50) | 0.715 | 0.398 |
| Total Bilirubin (umol/L) | 9.85 (7.80-14.88) | 9.35 (7.40-13.70) | 2.037 | 0.154 |
| Direct Bilirubin (umol/L) | 3.95 (3.08-5.60) | 4.15 (3.20-5.90) | 0.204 | 0.652 |
| Indirect Bilirubin (umol/L) | 6.60 (5.07-9.33) | 6.90 (4.60-8.90) | 0.516 | 0.473 |
| Total protein(g/L) | 65.85±6.50 | 65.47±6.62 | 0.29 | 0.591 |
| Albumin/Globulin(%) | 1.50 (1.30-1.70) | 1.50 (1.30-1.70) | 0.051 | 0.82 |
| Large platelet ratio(%) | 25.55 (20.28-31.72) | 24.55 (19.20-30.87) | 1.574 | 0.21 |
| Monocyte.percent(%) | 7.10 (5.90-8.10) | 6.70 (5.60-8.30) | 1.904 | 0.168 |
| Monocyte.count(10×10^9^/L) | 0.48 (0.37-0.65) | 0.50 (0.35-0.68) | 0.004 | 0.951 |
| Lymphocyte.percent(%) | 25.30 (15.95-34.00) | 25.25 (11.17-32.65) | 0.624 | 0.43 |
| Lymphocyte.count(10×109/L) | 1.73 (1.23-2.11) | 1.70 (1.31-2.11) | 0.154 | 0.695 |
| Thyroxine(pmol/L) | 100.80 (80.62-119.50) | 95.09 (80.28-116.30) | 0.048 | 0.826 |
| K(mmol/L) | 4.17 (3.83-4.41) | 4.10 (3.78-4.46) | 0.329 | 0.566 |
| Cl(mmol/L) | 107.35 (104.30-109.35) | 106.80 (103.82-108.90) | 0.867 | 0.352 |
| Na(mmol/L) | 141.40 (139.57-142.40) | 141.30 (139.90-142.80) | 0.381 | 0.537 |
| Mg(mmol/L) | 0.87 (0.80-0.93) | 0.89 (0.82-0.96) | 5.881 | 0.015 |
| Mean Platelet Volume (fL) | 9.80 (9.20-10.30) | 9.50 (9.00-10.00) | 5.341 | 0.021 |
| Globulin (g/L) | 27.25 (23.78-30.25) | 26.80 (23.50-30.60) | 0.025 | 0.874 |
| Basophil.percent(%) | 0.50 (0.30-0.60) | 0.50 (0.30-0.80) | 0.792 | 0.374 |
| Basophil.count(10×10^9^/L) | 0.03 (0.02-0.04) | 0.03 (0.02-0.05) | 2.514 | 0.113 |
| Free Thyroxine (pmol/L) | 16.79(14.67-18.89) | 16.68 (14.97-18.81) | <0.001 | 0.986 |
| Total bile acids (umol/L) | 4.00 (2.77-6.20) | 4.85 (3.00-7.80) | 4.567 | 0.033 |
| Platelet Distribution Width (%) | 16.00 (15.70-16.30) | 16.10 (15.80-16.40) | 1.801 | 0.18 |
| Procalcitonin (%) | 0.23 (0.19-0.29) | 0.23 (0.20-0.28) | 0.714 | 0.398 |
| Immature Granulocyte.percent(%) | 0.20 (0.10-0.60) | 0.20 (0.10-0.60) | 0.003 | 0.957 |
| Immature Granulocyte.count(fL) | 0.01 (0.01-0.04) | 0.02 (0.01-0.05) | 0.666 | 0.414 |
| Neutrophil.percent(%) | 72.75 (61.48-81.67) | 71.65 (61.65-82.40) | 0.13 | 0.719 |
| Neutrophil.count(fL) | 4.97 (3.45-7.42) | 5.25 (3.70-7.40) | 0.543 | 0.461 |
| rheumatoid factors(IU/ml) | 4.50 (2.00-11.25) | 4.50 (2.00-10.00) | 0.094 | 0.759 |
| dataset | 0.00 (0.00-0.00) | 1.00 (1.00-1.00) | 413 | <0.001 |
| sex |  |  | 0.497 | 0.481 |
| 1（male） | 40 (32.26%) | 104 (35.86%) |  |  |
| 2(female) | 84 (67.74%) | 186 (64.14%) |  |  |
| osteoporosis |  |  | 1.677 | 0.195 |
| 0 | 40 (32.26%) | 113 (38.97%) |  |  |
| 1 | 84 (67.74%) | 177 (61.03%) |  |  |
| nation |  |  | 8.459 | 0.489 |
| 1(Han) | 89 (71.77%) | 220 (75.86%) |  |  |
| 2(Zhuang) | 19 (15.32%) | 45 (15.52%) |  |  |
| 3(Yi) | 5 (4.03%) | 11 (3.79%) |  |  |
| 4(Miao) | 2 (1.61%) | 4 (1.38%) |  |  |
| 5(Yao) | 4 (3.23%) | 3 (1.03%) |  |  |
| 6(Hui) | 0 (0.00%) | 1 (0.34%) |  |  |
| 7(Tu Jia) | 3 (2.42%) | 5 (1.72%) |  |  |
| 13(MengGu) | 1 (0.81%) | 0 (0.00%) |  |  |
| 19(Bai) | 0 (0.00%) | 1 (0.34%) |  |  |
| History |  |  |  |  |
| smoking |  |  | 2.551 | 0.11 |
| 0 | 107 (86.29%) | 231 (79.66%) |  |  |
| 1 | 17 (13.71%) | 59 (20.34%) |  |  |
| drinking |  |  | 3.312 | 0.069 |
| 0 | 112 (90.32%) | 242 (83.45%) |  |  |
| 1 | 12 (9.68%) | 48 (16.55%) |  |  |
| hypertension |  |  | 0.016 | 0.899 |
| 0 | 71 (57.26%) | 168 (57.93%) |  |  |
| 1 | 53 (42.74%) | 122 (42.07%) |  |  |
| cerebral.infarction |  |  | 0.195 | 0.659 |
| 0 | 120 (96.77%) | 278 (95.86%) |  |  |
| 1 | 4 (3.23%) | 12 (4.14%) |  |  |
| encephalalatrophy |  |  | 0.027 | 0.869 |
| 0 | 116 (93.55%) | 270 (93.10%) |  |  |
| 1 | 8 (6.45%) | 20 (6.90%) |  |  |
| Geriatric.brain.changes |  |  | 0.429 | 0.513 |
| 0 | 124 (100.00%) | 289 (99.66%) |  |  |
| 1 | 0 (0.00%) | 1 (0.34%) |  |  |
| pneumonia |  |  | 0.166 | 0.683 |
| 0 | 116 (93.55%) | 268 (92.41%) |  |  |
| 1 | 8 (6.45%) | 22 (7.59%) |  |  |
| pnlmonary.nodule |  |  | 0.004 | 0.95 |
| 0 | 111 (89.52%) | 259 (89.31%) |  |  |
| 1 | 13 (10.48%) | 31 (10.69%) |  |  |
| hyperosteogeny |  |  | 0.033 | 0.855 |
| 0 | 122 (98.39%) | 286 (98.62%) |  |  |
| 1 | 2 (1.61%) | 4 (1.38%) |  |  |
| atherosclerosis |  |  | 0.05 | 0.823 |
| 0 | 121 (97.58%) | 284 (97.93%) |  |  |
| 1 | 3 (2.42%) | 6 (2.07%) |  |  |
| rheumatoid.arthritis |  |  | 0.463 | 0.496 |
| 0 | 119 (95.97%) | 282 (97.24%) |  |  |
| 1 | 5 (4.03%) | 8 (2.76%) |  |  |
| fracture |  |  | 2.393 | 0.122 |
| 0 | 95 (76.61%) | 241 (83.10%) |  |  |
| 1 | 29 (23.39%) | 49 (16.90%) |  |  |

Baseline characteristics are presented as mean (SD), median (IQR), or n (%). Percentages might not total 100 because of rounding. Continuous variables were compared using Student's t-test (normally distributed data) or Mann-Whitney U test (non-normally distributed data); categorical variables were analyzed by Chi-square test or Fisher's exact test. P < 0.01 vs. control group. 0=negative, 1=positive.
