# Supplementary material for: A novel nomogram for predicting osteoporosis with low back pain among the patients in Wenshan Zhuang and Miao Autonomous Prefecture of China
Source: Front Endocrinol (Lausanne). 2025 Jun 5;16:1535163. doi: 10.3389/fendo.2025.1535163 (PMC12176570; doi:10.3389/fendo.2025.1535163)
Supplement: Supplementary file 3 [file Table3.docx]

**TableS3. Univariate Logistic Regression Analysis of Predictors for Osteoporosis**

**in Low Back Pain Patients(backward selection method)**

| Characteristics | β | Se | OR | CI(95%) | Z | P |
| --- | --- | --- | --- | --- | --- | --- |
| Systolic blood pressure(mmHg) | 0.004 | 0.005 | 1 | 0.99-1.01 | 0.815 | 0.415 |
| Diastolic blood pressure(mmHg) | 0.001 | 0.009 | 1 | 0.98-1.02 | 0.14 | 0.889 |
| age,years | 0.081 | 0.012 | 1.08 | 1.06-1.11 | 6.787 | 0 |
| sex | 0.717 | 0.251 | 2.05 | 1.25-3.35 | 2.862 | 0.004 |
| nation2(Zhuang) | 0 | 0.334 | 1 | 0.52-1.92 | 0 | 1 |
| nation3(Yi) | 17.161 | 1192.833 | 28363208.23 | 0-Inf | 0.014 | 0.989 |
| nation4(Miao) | 17.161 | 1978.09 | 28363208.23 | 0-Inf | 0.009 | 0.993 |
| nation5(Yao) | -17.972 | 2284.102 | 0 | 0-Inf | -0.008 | 0.994 |
| nation6(Hui) | 17.161 | 3956.18 | 28363208.22 | 0-Inf | 0.004 | 0.997 |
| nation7(Tu Jia) | -0.811 | 0.923 | 0.44 | 0.07-2.71 | -0.878 | 0.38 |
| nation19(Bai) | -17.972 | 3956.18 | 0 | 0-Inf | -0.005 | 0.996 |
| smoking | 0.464 | 0.313 | 1.59 | 0.86-2.94 | 1.485 | 0.138 |
| drinking | 0.896 | 0.367 | 2.45 | 1.19-5.03 | 2.441 | 0.015 |
| hypertension | 0.454 | 0.248 | 1.58 | 0.97-2.56 | 1.834 | 0.067 |
| cerebral.infarction | 1.201 | 0.784 | 3.32 | 0.71-15.45 | 1.531 | 0.126 |
| encephalalatrophy | 1.36 | 0.638 | 3.9 | 1.12-13.6 | 2.13 | 0.033 |
| Geriatric.brain.changes | -15.024 | 882.743 | 0 | 0-Inf | -0.017 | 0.986 |
| pneumonia | -0.088 | 0.451 | 0.92 | 0.38-2.22 | -0.194 | 0.846 |
| pnlmonary.nodule | 0.865 | 0.448 | 2.38 | 0.99-5.72 | 1.932 | 0.053 |
| hyperosteogeny | -0.455 | 1.007 | 0.63 | 0.09-4.57 | -0.452 | 0.651 |
| atherosclerosis | 1.18 | 1.102 | 3.26 | 0.38-28.23 | 1.071 | 0.284 |
| rheumatoid.arthritis | 16.164 | 848.367 | 10465111.06 | 0-Inf | 0.019 | 0.985 |
| fracture | 3.73 | 1.019 | 41.67 | 5.66-307.09 | 3.662 | 0 |
| Totalcholesterol  (mmol/L) | -0.431 | 0.105 | 0.65 | 0.53-0.8 | -4.103 | 0 |
| Triglyceride  (mmol/L) | -0.185 | 0.095 | 0.83 | 0.69-1 | -1.938 | 0.053 |
| High Density Lipoprotein (mmol/L) | -0.394 | 0.312 | 0.67 | 0.37-1.24 | -1.263 | 0.207 |
| Low Density Lipoprotein (mmol/L) | -0.46 | 0.135 | 0.63 | 0.48-0.82 | -3.404 | 0.001 |
| Creative kinaseisoenzyme MB(ng/ml) | -0.002 | 0.007 | 1 | 0.98-1.01 | -0.251 | 0.802 |
| C-reactive protein(mg/L) | 0.013 | 0.005 | 1.01 | 1-1.02 | 2.772 | 0.006 |
| Ca(mmol/L) | -1.052 | 1.001 | 0.35 | 0.05-2.48 | -1.051 | 0.293 |
| procalcitonin(ng/ml) | -0.018 | 0.029 | 0.98 | 0.93-1.04 | -0.615 | 0.539 |
| Uric Acid (umol/L) | 0 | 0.001 | 1 | 1-1 | -0.052 | 0.959 |
| Hemoglobin (g/L) | -0.041 | 0.007 | 0.96 | 0.95-0.97 | -5.935 | 0 |
| Glucose (g/L) | 0.072 | 0.072 | 1.07 | 0.93-1.24 | 1.001 | 0.317 |
| Fibrinogen(g/L) | 0.121 | 0.088 | 1.13 | 0.95-1.34 | 1.367 | 0.172 |
| D.dimer(mg/L) | 0.137 | 0.055 | 1.15 | 1.03-1.28 | 2.467 | 0.014 |
| γ-Glutamyl Transferase (U/L) | 0 | 0.002 | 1 | 1-1 | -0.095 | 0.925 |
| Alanine Aminotransferase (U/L) | -0.021 | 0.007 | 0.98 | 0.97-0.99 | -3.04 | 0.002 |
| Aspartate Aminotransferase (U/L) | 0.006 | 0.007 | 1.01 | 0.99-1.02 | 0.905 | 0.366 |
| Albumin (U/L) | -0.12 | 0.031 | 0.89 | 0.83-0.94 | -3.866 | 0 |
| White Blood Cell (g/L) | 0.014 | 0.025 | 1.01 | 0.97-1.06 | 0.538 | 0.59 |
| Red Blood Cell (g/L) | -1.278 | 0.228 | 0.28 | 0.18-0.44 | -5.611 | 0 |
| Packed Cell Volume (fL) | -0.169 | 0.028 | 0.84 | 0.8-0.89 | -5.948 | 0 |
| Creative kinase(ng/ml) | 0 | 0 | 1 | 1-1 | 0.492 | 0.623 |
| Creatinine(umol/L) | 0.002 | 0.002 | 1 | 1-1.01 | 1.051 | 0.293 |
| Alkaline phosphatase(U/L) | 0.004 | 0.003 | 1 | 1-1.01 | 1.587 | 0.112 |
| Urea nitrogen(mmol/L) | -0.018 | 0.027 | 0.98 | 0.93-1.04 | -0.677 | 0.498 |
| Mean Corpuscular Volume (fL) | 0.004 | 0.015 | 1 | 0.98-1.03 | 0.268 | 0.789 |
| Mean Corpuscular Hemoglobin (pg) | -0.006 | 0.042 | 0.99 | 0.92-1.08 | -0.137 | 0.891 |
| Mean Corpuscular Hemoglobin Concentration (g/L) | -0.01 | 0.012 | 0.99 | 0.97-1.01 | -0.824 | 0.41 |
| Lactate Dehydrogenase (U/L) | 0.002 | 0.001 | 1 | 1-1 | 1.536 | 0.125 |
| Platelet Count (10×109/L) | 0.001 | 0.001 | 1 | 1-1 | 1.081 | 0.28 |
| Total Bilirubin (umol/L) | 0 | 0.014 | 1 | 0.97-1.03 | -0.031 | 0.976 |
| Direct Bilirubin (umol/L) | 0.118 | 0.054 | 1.12 | 1.01-1.25 | 2.18 | 0.029 |
| Indirect Bilirubin (umol/L) | -0.033 | 0.028 | 0.97 | 0.92-1.02 | -1.169 | 0.242 |
| Total protein(g/L) | -0.032 | 0.018 | 0.97 | 0.93-1 | -1.75 | 0.08 |
| Albumin/Globulin(%) | -1.026 | 0.4 | 0.36 | 0.16-0.79 | -2.564 | 0.01 |
| Large platelet ratio(%) | 0.011 | 0.014 | 1.01 | 0.98-1.04 | 0.784 | 0.433 |
| Monocyte.percent(%) | 0.13 | 0.06 | 1.14 | 1.01-1.28 | 2.171 | 0.03 |
| Monocyte.count(10×10^9^/L) | 0.633 | 0.475 | 1.88 | 0.74-4.78 | 1.333 | 0.183 |
| Lymphocyte.percent(10×109/L) | -0.017 | 0.01 | 0.98 | 0.96-1 | -1.799 | 0.072 |
| Lymphocyte.count(10×109/L) | -0.537 | 0.169 | 0.58 | 0.42-0.81 | -3.175 | 0.001 |
| Thyroxine(pmol/L) | -0.001 | 0.005 | 1 | 0.99-1.01 | -0.224 | 0.823 |
| K(mmol/L) | 0.465 | 0.217 | 1.59 | 1.04-2.44 | 2.141 | 0.032 |
| Cl(mmol/L) | -0.134 | 0.031 | 0.87 | 0.82-0.93 | -4.279 | 0 |
| Na(mmol/L) | 0.006 | 0.042 | 1.01 | 0.93-1.09 | 0.137 | 0.891 |
| Mg(mmol/L) | 0.611 | 0.948 | 1.84 | 0.29-11.81 | 0.644 | 0.519 |
| Mean Platelet Volume (fL) | -0.067 | 0.106 | 0.93 | 0.76-1.15 | -0.634 | 0.526 |
| Globulin (g/L) | 0.051 | 0.024 | 1.05 | 1-1.1 | 2.139 | 0.032 |
| Basophil.percent(%) | 0.121 | 0.287 | 1.13 | 0.64-1.98 | 0.422 | 0.673 |
| Basophil.count(10×10^9^/L) | 1.734 | 2.922 | 5.66 | 0.02-1739.52 | 0.593 | 0.553 |
| Free Thyroxine (pmol/L) | 0.029 | 0.033 | 1.03 | 0.97-1.1 | 0.882 | 0.378 |
| Total bile acids (umol/L) | 0.003 | 0.011 | 1 | 0.98-1.02 | 0.262 | 0.793 |
| Platelet Distribution Width (%) | -0.128 | 0.079 | 0.88 | 0.75-1.03 | -1.609 | 0.108 |
| Procalcitonin (%) | 1.601 | 1.331 | 4.96 | 0.37-67.33 | 1.203 | 0.229 |
| Immature Granulocyte. percent(%) | 0.082 | 0.114 | 1.09 | 0.87-1.36 | 0.721 | 0.471 |
| Immature Granulocyte.count(fL) | 0.468 | 0.602 | 1.6 | 0.49-5.19 | 0.777 | 0.437 |
| Neutrophil.percent(%) | 0.018 | 0.009 | 1.02 | 1-1.04 | 1.979 | 0.048 |
| Neutrophil.count(fL) | 0.041 | 0.046 | 1.04 | 0.95-1.14 | 0.881 | 0.378 |
| Rheumatoid factors(IU/ml) | 0.014 | 0.006 | 1.01 | 1-1.03 | 2.103 | 0.035 |

coefficients (β) standard errors (SE), Odds ratio(OR), P-values were derived from linear regression, Significance level based on Z value（P）*P < 0.05,95% confidence interval for OR(CI).
